# Supplementary material for: Control of polymers’ amorphous-crystalline transition enables miniaturization and multifunctional integration for hydrogel bioelectronics
Source: Nat Commun. 2024 Apr 25;15:3525. doi: 10.1038/s41467-024-47988-w (PMC11045824; doi:10.1038/s41467-024-47988-w)
Supplement: Supplementary file 1 — Supplementary Information [file 41467_2024_47988_MOESM1_ESM.pdf]

Supplementary Information

This supplementary information contains:

Supplementary Discussion:

Supplementary Figures 1-44, Supplementary Tables 1-5

Supplementary References

Supplementary Discussion

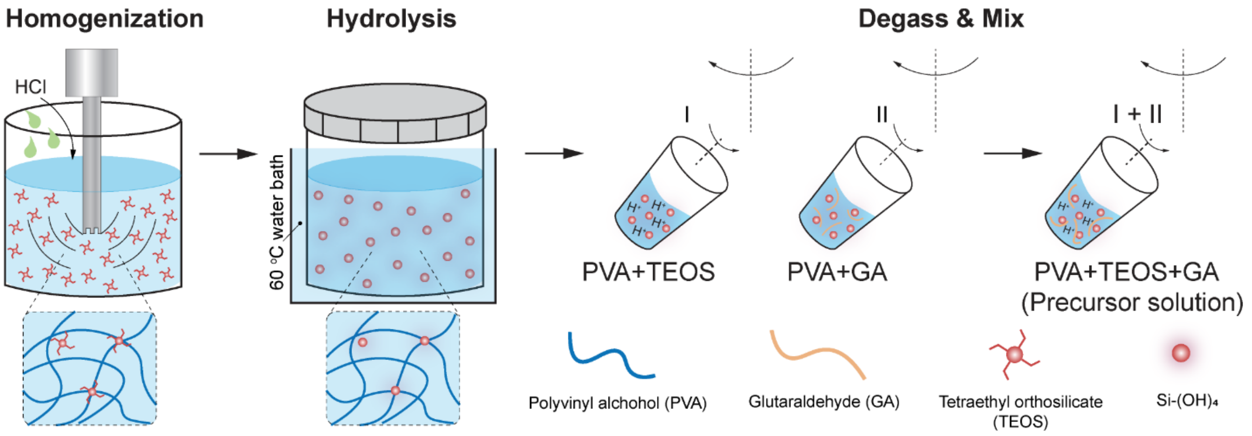

Supplementary Figure 1. COMPACT hydrogel synthesis. Tetraethyl orthosilicate (TEOS) was incorporated into a 10 wt.% poly(vinyl) alcohol (PVA) solution, followed by two different levels of homogenization. The homogenization process resulted in the formation of an oil-water phase in PVA-TEOS, and diluted hydrochloric acid HCl solutions were gradually added during the process. TEOS underwent gradual hydrolysis into $Si(OH)_4$ groups at 60 °C for 12 hours with HCl acting as a catalyst. The resulting PVA-TEOS solution was then degassed and mixed in a planetary mixer. Glutaraldehyde (GA) was added into a 10 wt.% PVA solution, followed by degassing and mixing. The two mixed solutions were combined (at a weight ratio of 1:1), degassed, and mixed to form precursor solutions for later use. The planetary mixer was operated with consistent mixing parameters of 2000 rpm, 1 minute, and a vacuum of 16 kPa.

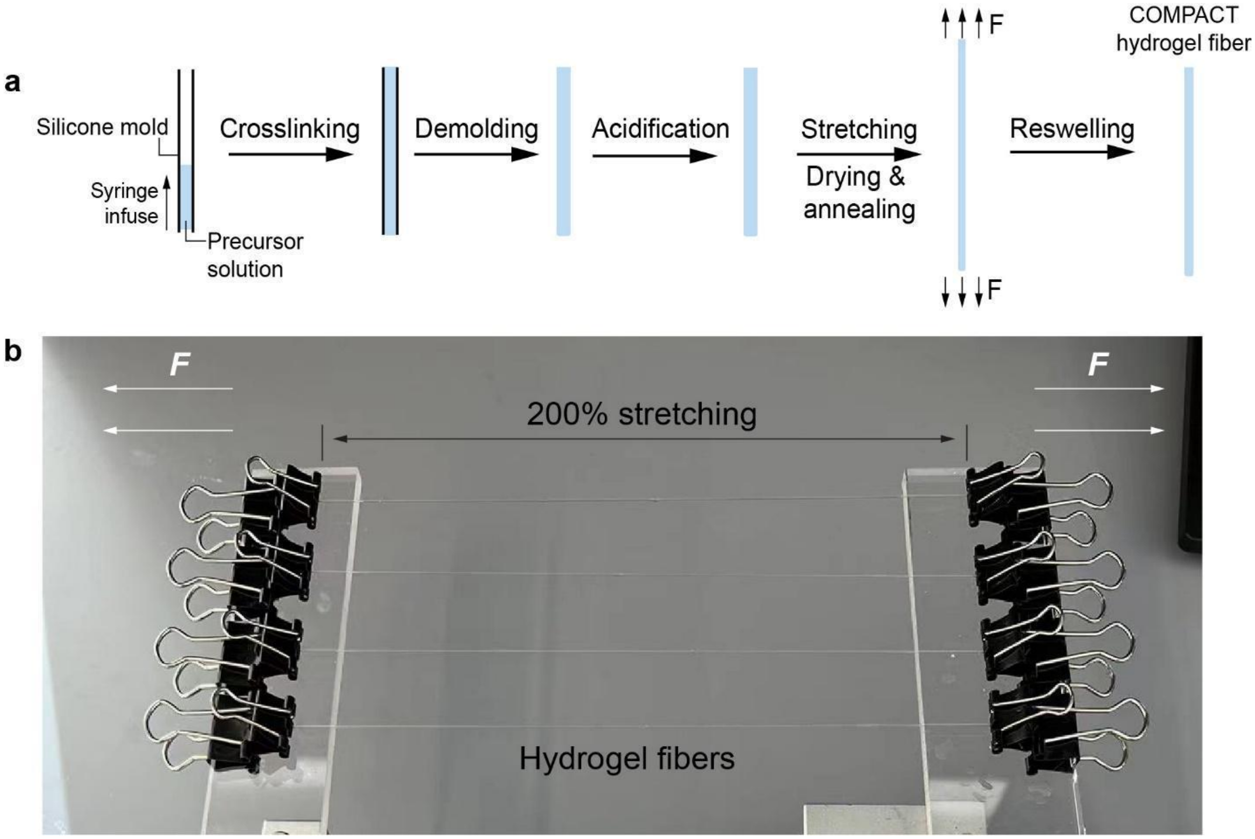

Supplementary Figure 2. COMPACT hydrogel fiber fabrication. a, The precursor solution (**Supplementary Figure 1**) was introduced into a silicone mold (300-800 μm) and allowed to undergo cross-linking at room temperature (RT) for 4 hours, resulting in a fiber shape. Subsequently, the silicone mold containing the cross-linked PVA hydrogel was soaked in dichloromethane (DCM) for 5 minutes to induce mold swelling. The resulting cross-linked PVA hydrogel fiber was then extracted from the mold and immersed in 12 mM HCl solution for 2 hours. The acidified PVA hydrogel fiber was stretched and dried at 60 ^{o}C for 12 hours and annealed at 100 ^{o}C for 20 minutes. Finally, the annealed PVA hydrogel fiber was swollen in water to obtain the COMPACT hydrogel fiber. **b,** Stretching demonstration of hydrogel fibers. A customized stretching device for hydrogel fibers stretching (200% stretching).

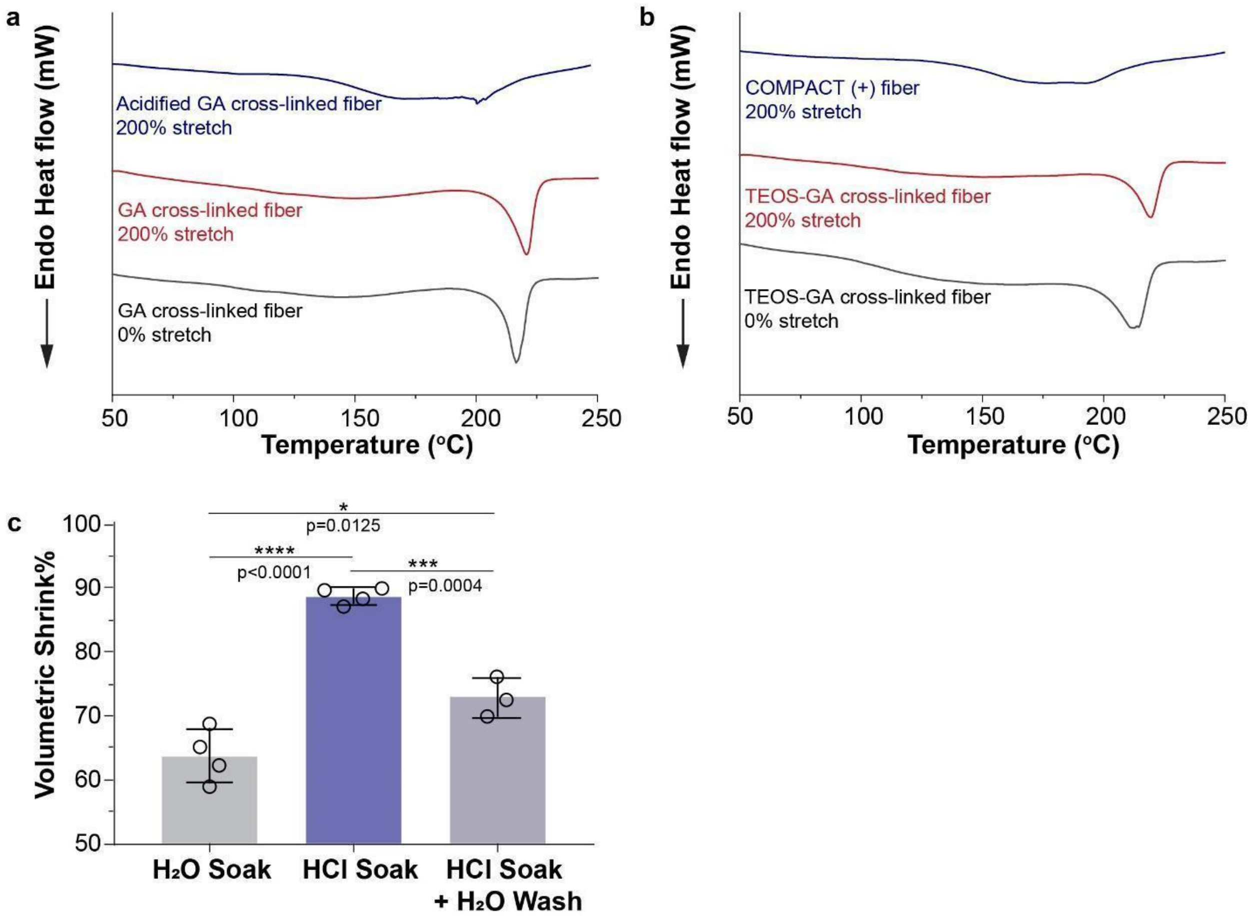

Supplementary Figure 3. Characterization of crystallinity in PVA hydrogels. a, Representative differential scanning calorimetry (DSC) thermographs of 0% stretch GA cross-linked fibers, 200% stretch GA cross-linked fibers, and 200% stretched and acidified GA cross-linked fibers. **b**, Representative DSC thermographs of 0% stretch TEOS-GA cross-linked fibers, 200% stretch TEOS-GA cross-linked fibers, and COMPACT (+) cross-linked fibers. **c**, Influence of acidification treatment on polymeric crystallinity. Acidified samples significantly increase the volumetric shrinkage compared to untreated groups. Groups with acidification followed by water wash show a significant decrease in the volumetric shrinkage (One-way ANOVA and Tukey's multiple comparisons tests, $F_{2,\ 8}$=66.05, ****$p<0.0001$). Data presented as mean ± standard deviation (s.d.), n=3-4 individual hydrogel fibers.

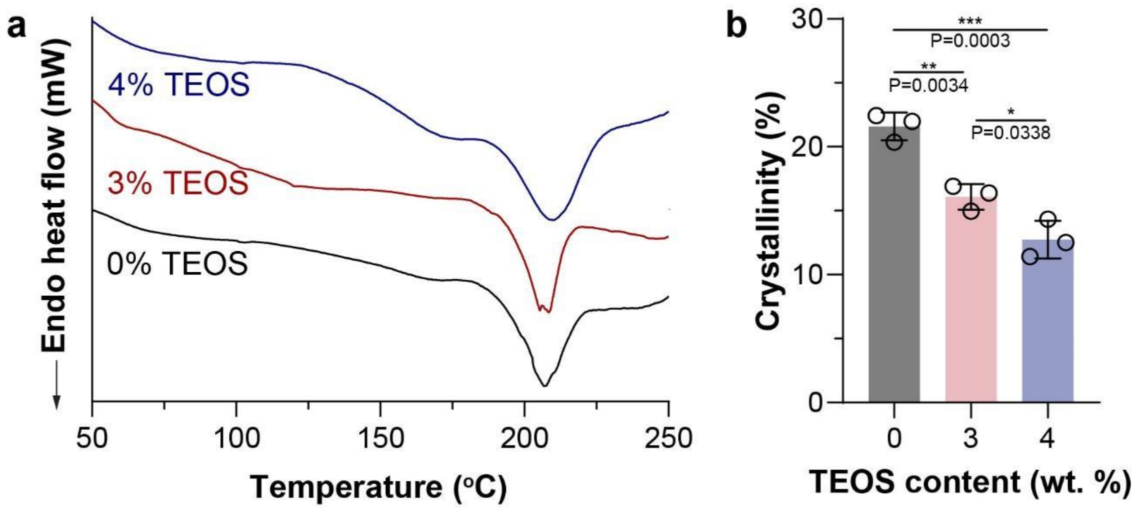

Supplementary Figure 4. Characterization of crystallinity in COMPACT hydrogels. a, Representative DSC profiles of COMPACT hydrogel fibers including different TEOS contents (0, 3% and 4%, with acidification and 200% stretching). **b**, Crystallinity (%) of COMPACT hydrogel fibers calculated from **a** (One-way ANOVA and Tukey's multiple comparisons tests, $F_{2, 6}$=41.12, ***p=0.0003). Mean ± s.d., n=3 independent hydrogel fibers.

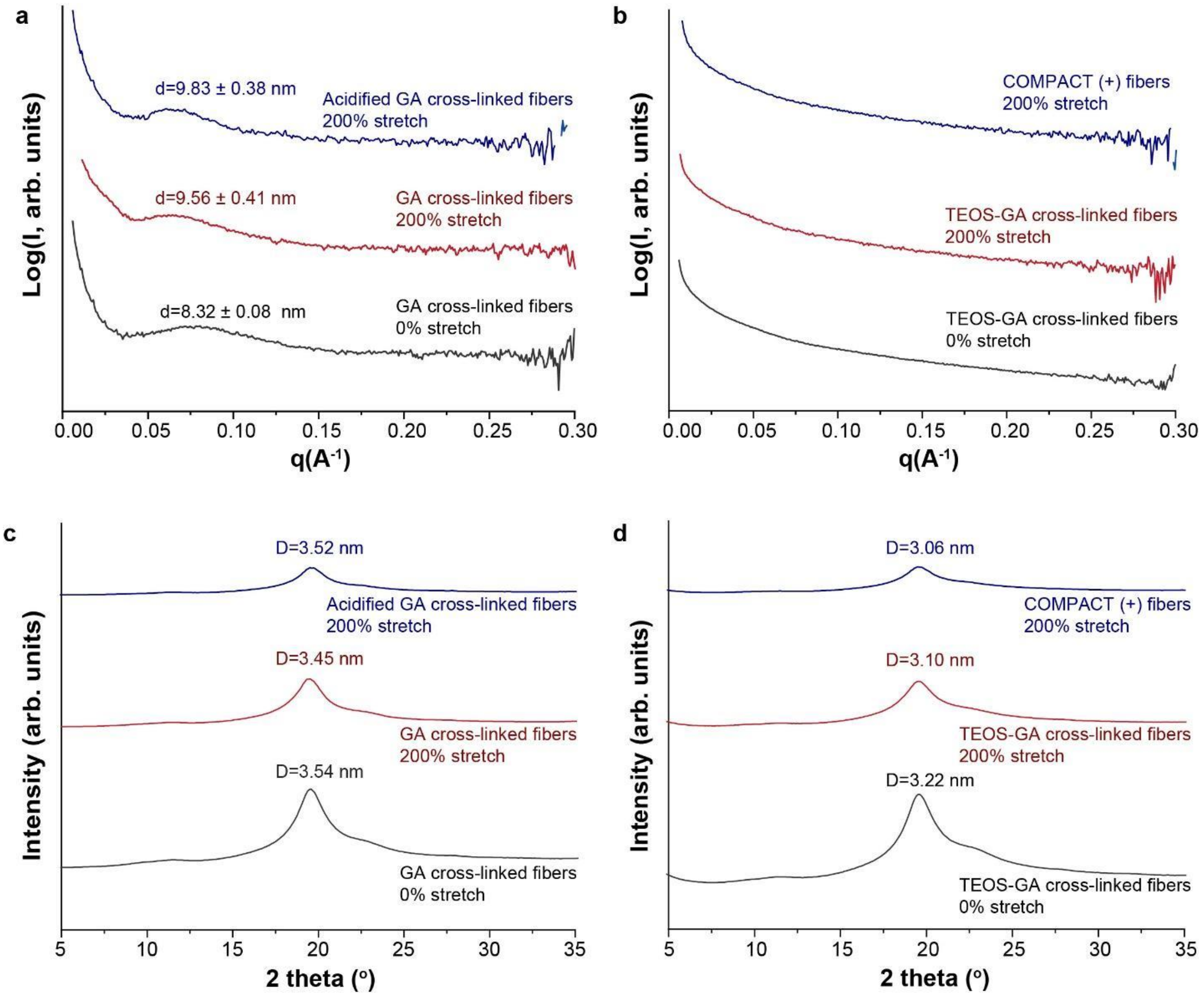

Supplementary Figure 5. Structural characterization of nanocrystalline domains in PVA hydrogels. a, Representative Small-angle X-ray Scattering (SAXS) profiles of 0% stretch GA cross-linked fibers, 200% stretch GA cross-linked fibers, and 200% stretched and acidified GA cross-linked fibers. d: spacing between nanocrystalline domains. **b**, Representative SAXS profiles of 0% stretch TEOS-GA cross-linked fibers, 200% stretch TEOS-GA cross-linked fibers, and COMPACT (+) cross-linked fibers. **c**, Representative Wide-angle X-ray Scattering (WAXS) profiles of 0% stretch GA cross-linked fibers, 200% stretch GA cross-linked fibers, and 200% stretched and acidified GA cross-linked fibers. D: the size of nanocrystalline domains. a.u.: arbitrary units. **d**, Representative WAXS profiles of 0% stretch TEOS-GA cross-linked fibers, 200% stretch TEOS-GA cross-linked fibers, and COMPACT (+) cross-linked fibers. D: the size of nanocrystalline domains. arb.units.: arbitrary units.

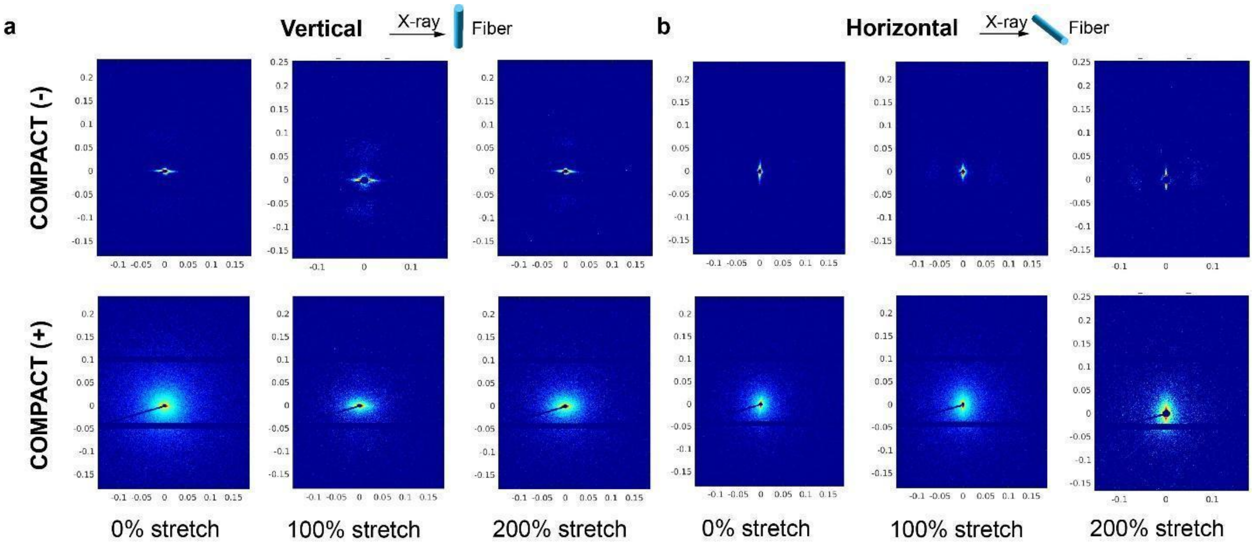

Supplementary Figure 6. SAXS two-dimensional (2D) spectra of nanocrystalline domain orientations in hydrogel fibers. **a**, Representative 2-dimensional SAXS spectra (vertical measurement) collected from 0% stretch, 100% stretch, 200% stretch COMPACT (-), and COMPACT (+) hydrogels, respectively. **b**, Representative 2-dimensional SAXS spectra (horizontal measurement) collected from 0% stretch, 100% stretch, 200% stretch COMPACT (-), and COMPACT (+) hydrogels, respectively.

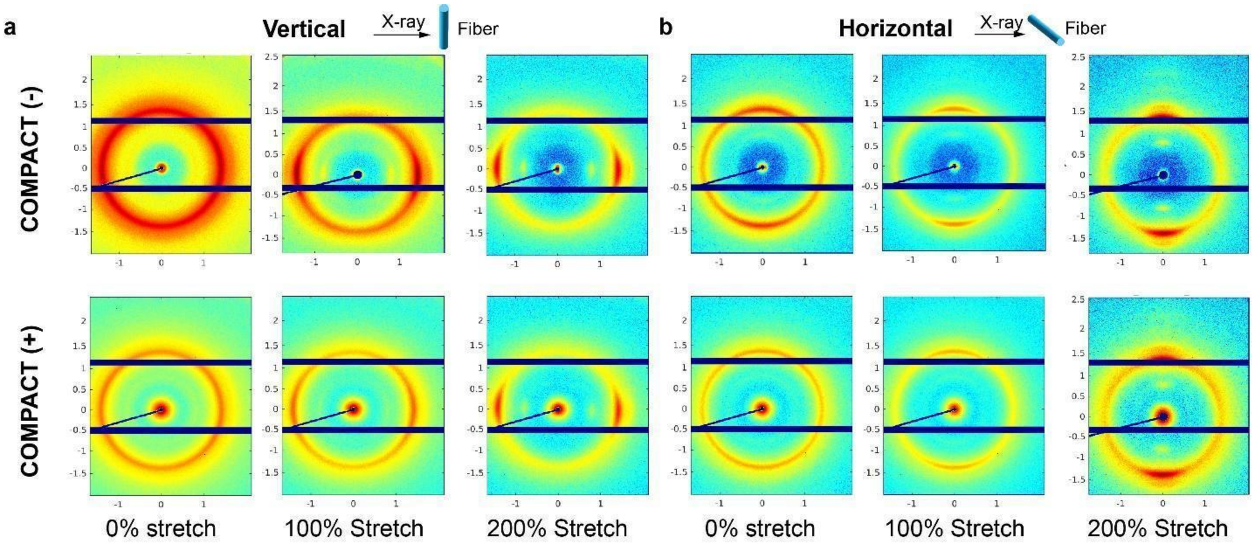

Supplementary Figure 7. Wide-angle X-ray Scattering (WAXS) 2D spectra of nanocrystalline domain orientations in hydrogel fibers. **a**, Representative 2-dimensional WAXS spectra (vertical measurement) collected from 0% stretch, 100% stretch, 200% stretch COMPACT (-), and COMPACT (+) hydrogels, respectively. **b**, Representative 2-dimensional WAXS spectra (horizontal measurement) collected from 0% stretch, 100% stretch, 200% stretch COMPACT (-), and COMPACT (+) hydrogels, respectively. Each hydrogel fiber was repeated three times with similar results.

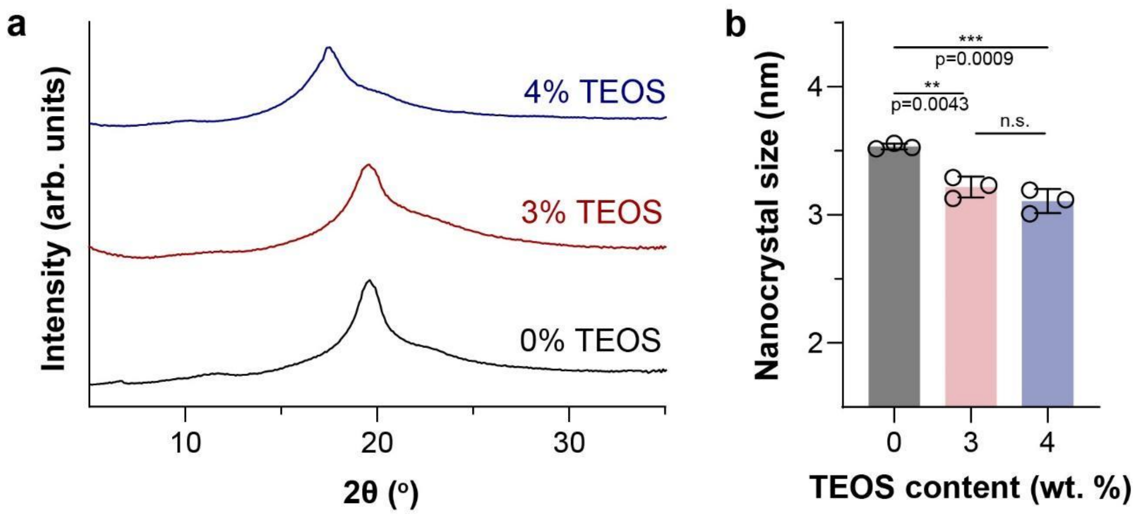

Supplementary Figure 8. Characterization of nanocrystal size in COMPACT hydrogels. a, Representative WAXS curves of COMPACT hydrogel fibers including different TEOS contents (0, 3% and 4%, with acidification and 200% stretching). **b**, Nanocrystal size of COMPACT hydrogel fibers calculated from **a** (One-way ANOVA and Tukey's multiple comparisons tests, $F_{2, 6}=27.76$, ***p=0.0009). Mean ± s.d., n=3 independent hydrogel fibers.

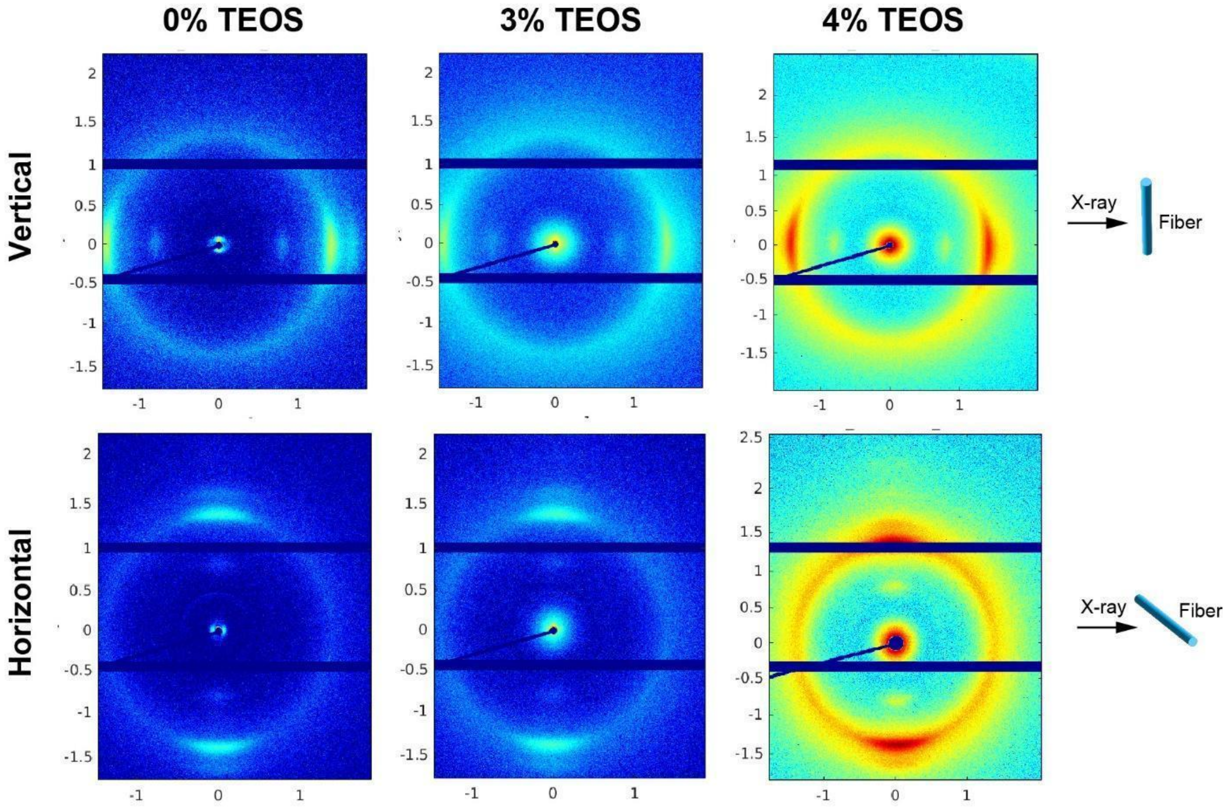

Supplementary Figure 9. WAXS 2D patterns of nanocrystalline domain orientations in COMPACT hydrogels. Representative WAXS 2D patterns (vertical and horizontal measurements) of COMPACT hydrogel fibers including different TEOS contents (0, 3% and 4%, with acidification and 200% stretching).

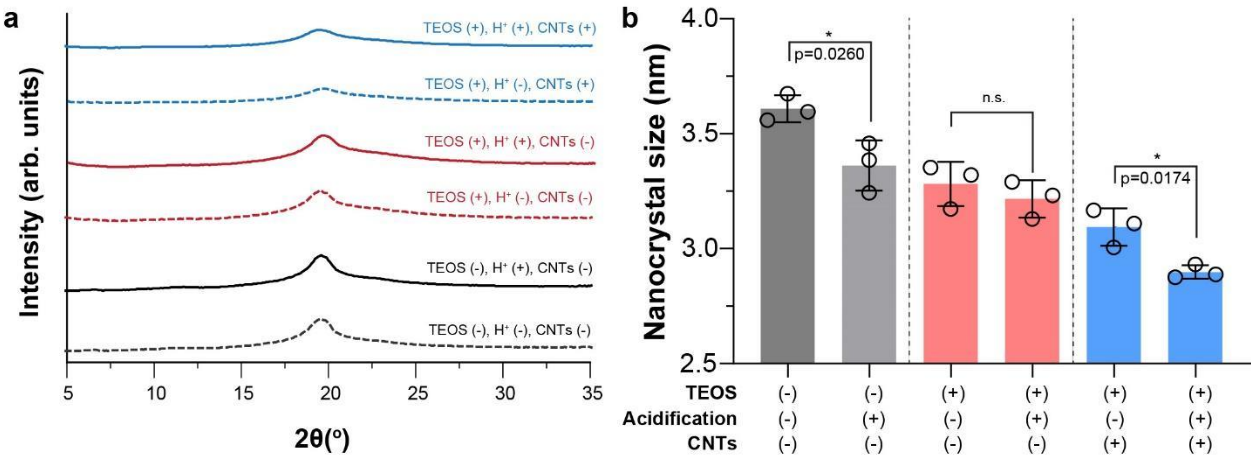

Supplementary Figure 10. The effect of acidification on nanocrystal size. a, WAXS curves of different sample groups with or without acidification treatment. Group 1: TEOS (-) and CNTs (-) with and without acidification; Group 2: TEOS (+) and CNTs (-) with and without acidification; Group 3: TEOS (+) and CNTs (+) with and without acidification. **b**, Calculated nanocrystal size from **a** (Two-tailed unpaired student's t-test. Group 1: F=3.448, t=3.454, df=4, *p=0.0260. Group 2: F=1.398, t=0.8935, df=4, n.s. p=0.4221. Group 3: F=7.508, t=3.911, df=4, *p=0.0174). Mean ± s.d., n=3 independent hydrogel fibers.

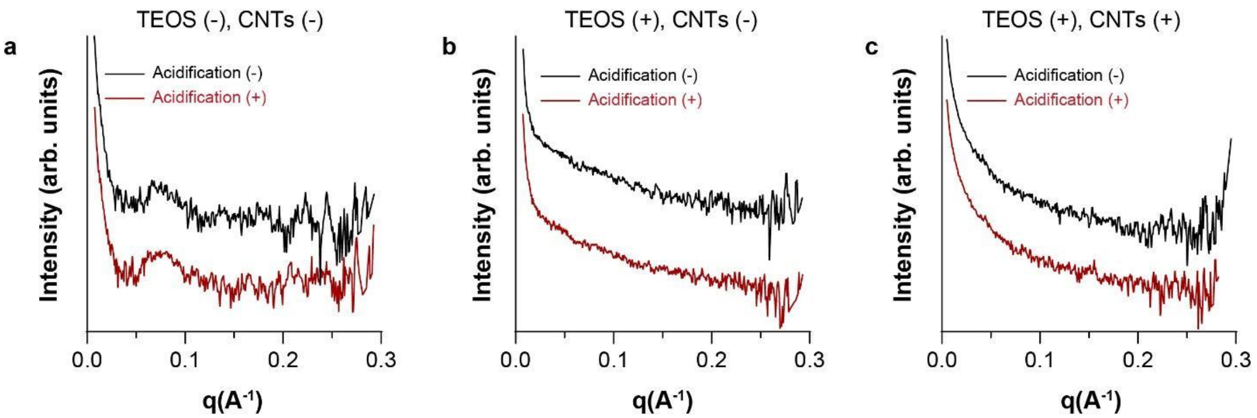

Supplementary Figure 11. The effect of acidification on nanocrystalline domain spacing. a, Representative SAXS profiles collected from hydrogel fibers (TEOS (-) and CNTs (-) with and without acidification). **b**, Representative SAXS profiles collected from hydrogel fibers (TEOS (+) and CNTs (-) with and without acidification). **c**, Representative SAXS profiles collected from hydrogel fibers (TEOS (+) and CNTs (+) with and without acidification). Each hydrogel fiber was repeated three times with similar results.

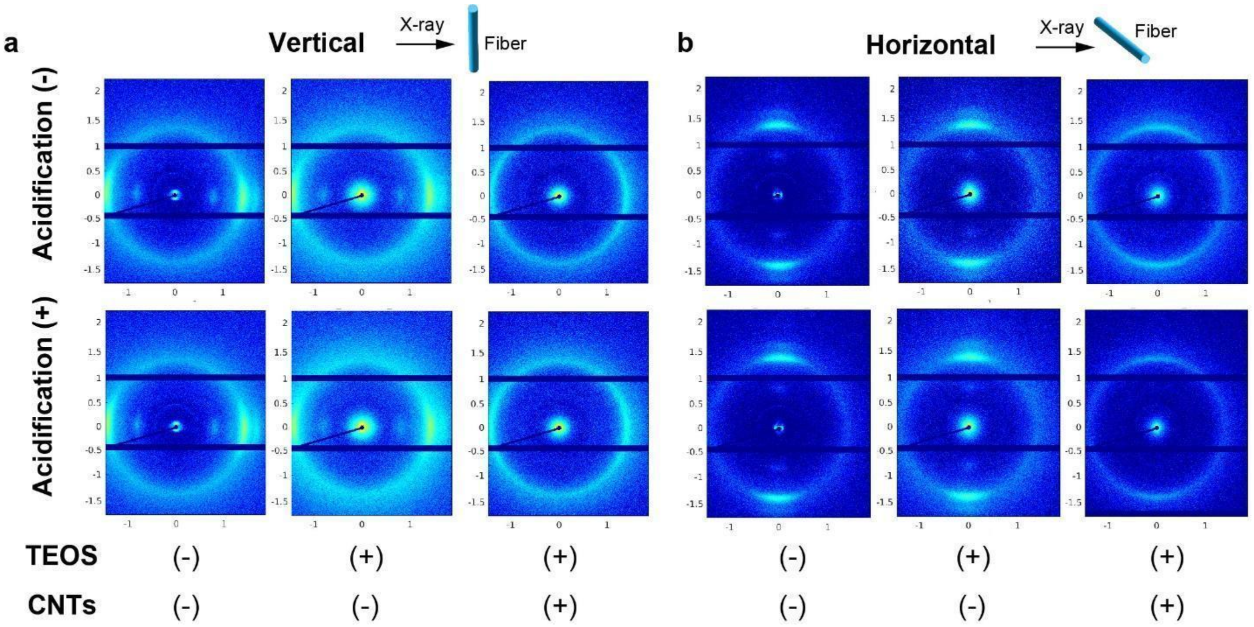

Supplementary Figure 12. WAXS 2D patterns of nanocrystalline domain orientations in COMPACT hydrogels. a, Representative 2D WAXS spectra (vertical measurement) collected from PVA hydrogel fibers (groups of TEOS (-) and CNTs (-), TEOS (+) and CNTs (-), and TEOS (+) and CNTs (+)) with and without acidification. **b,** Representative 2D WAXS spectra (horizontal measurement) collected from PVA hydrogel fibers (groups of TEOS (-) and CNTs (-), TEOS (+) and CNTs (-), and TEOS (+) and CNTs (+)) with and without acidification.

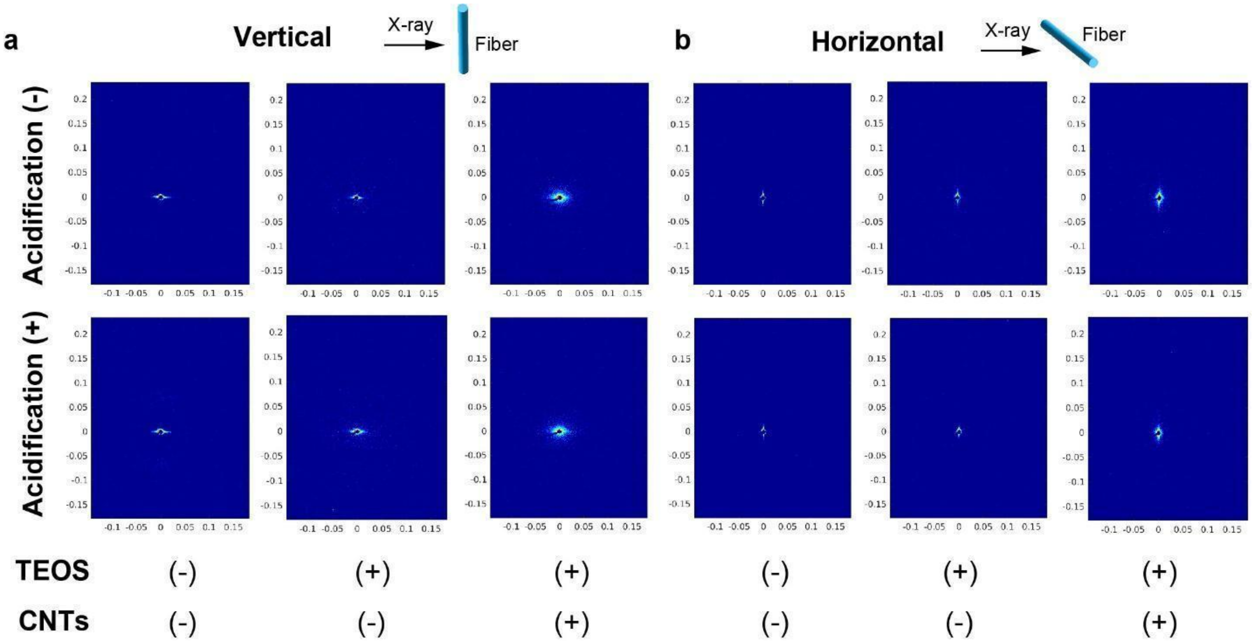

Supplementary Figure 13. SAXS 2D patterns of nanocrystalline domain orientations in COMPACT hydrogels. a, Representative 2D SAXS spectra (vertical measurement) collected from PVA hydrogel fibers (groups of TEOS (-) and CNTs (-), TEOS (+) and CNTs (-), and TEOS (+) and CNTs (+)) with and without acidification. **b,** Representative 2D SAXS spectra (horizontal measurement) collected from PVA hydrogel fibers (groups of TEOS (-) and CNTs (-), TEOS (+) and CNTs (-), and TEOS (+) and CNTs (+)) with and without acidification.

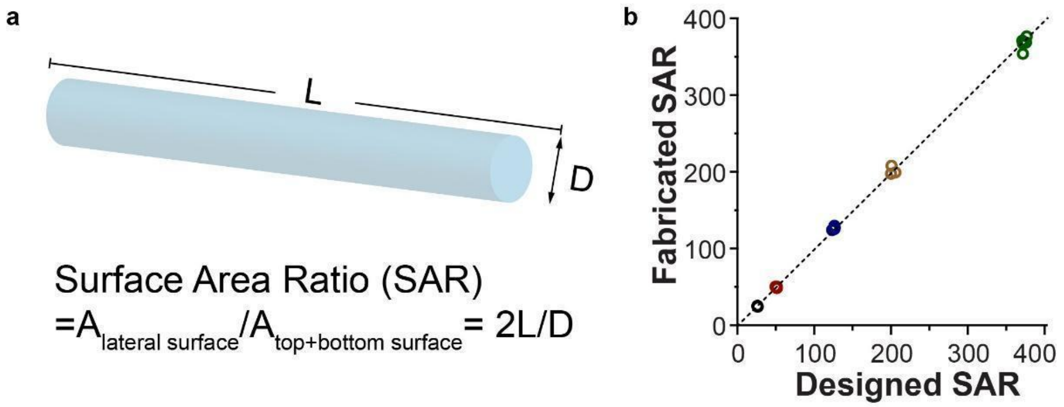

Supplementary Figure 14. Calibration of fiber molding and extrusion fabrication method. a, Schematic illustration of fiber surface area ratio (SAR). **b**, Relation between fabricated fiber SAR and mold SAR showing good agreements (R^2=0.9995). Each dot represents one independent fiber sample.

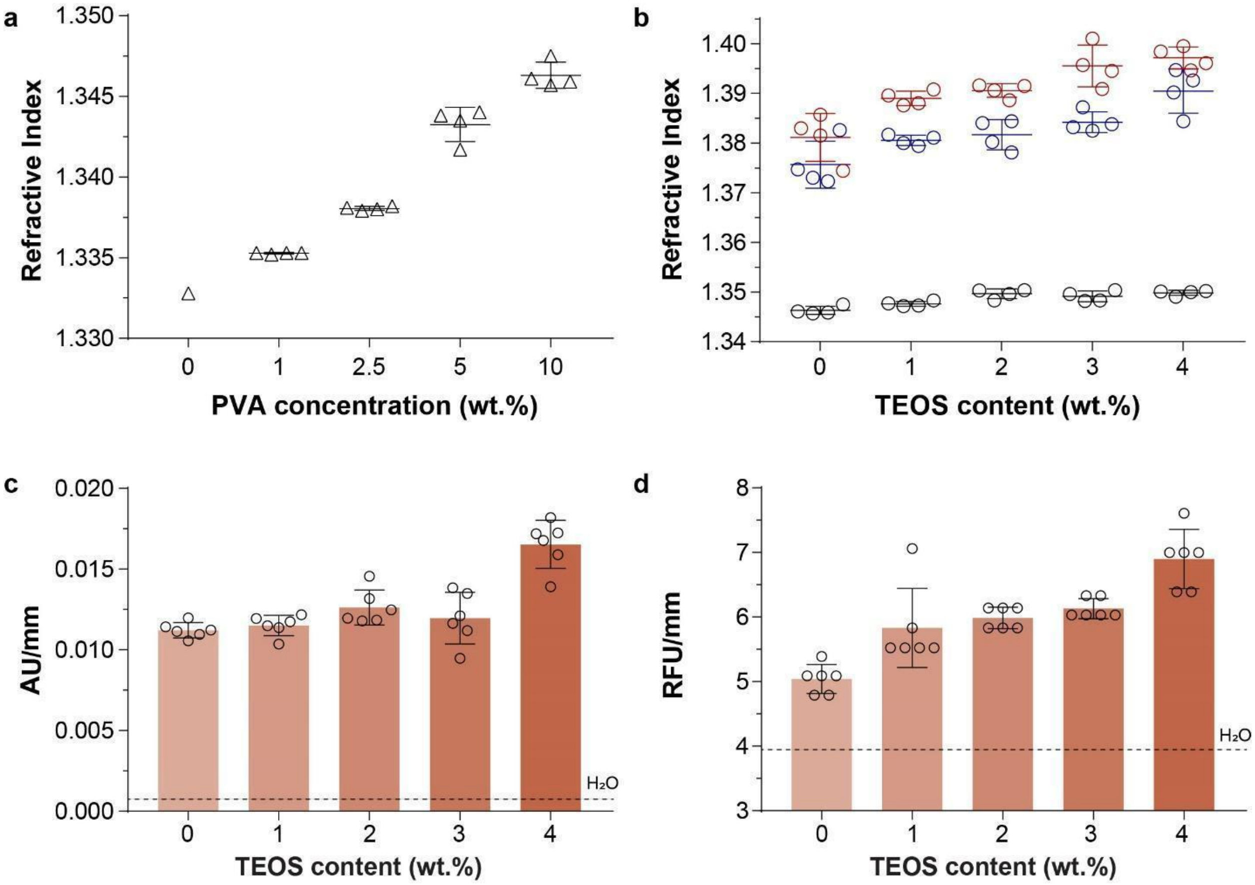

Supplementary Figure 15. The optical performance of COMPACT hydrogel membranes. a, Relation between refractive index and polymer concentration that follows the rule of mixtures (original state). **b**, Refractive index measurements of hydrogels with different TEOS contents (black: original samples, blue: rehydrated TEOS-GA cross-linked samples, red: acidified TEOS-GA cross-linked samples under rehydrated state**)**. **c,** Absorbance values of COMPACT hydrogels with different TEOS contents. The measured absorbance values were normalized to the sample thickness and 0 wt.% TEOS samples were used as a reference. AU: absorbance units. **d**, Autofluorescence values of COMPACT hydrogels with different TEOS contents. The measured absorbance values were normalized to the sample thickness and 0 wt.% TEOS samples were used as a reference. RFU: relative fluorescence units. All the plots are presented as mean ± s.d., n=4-6 independent hydrogel fibers.

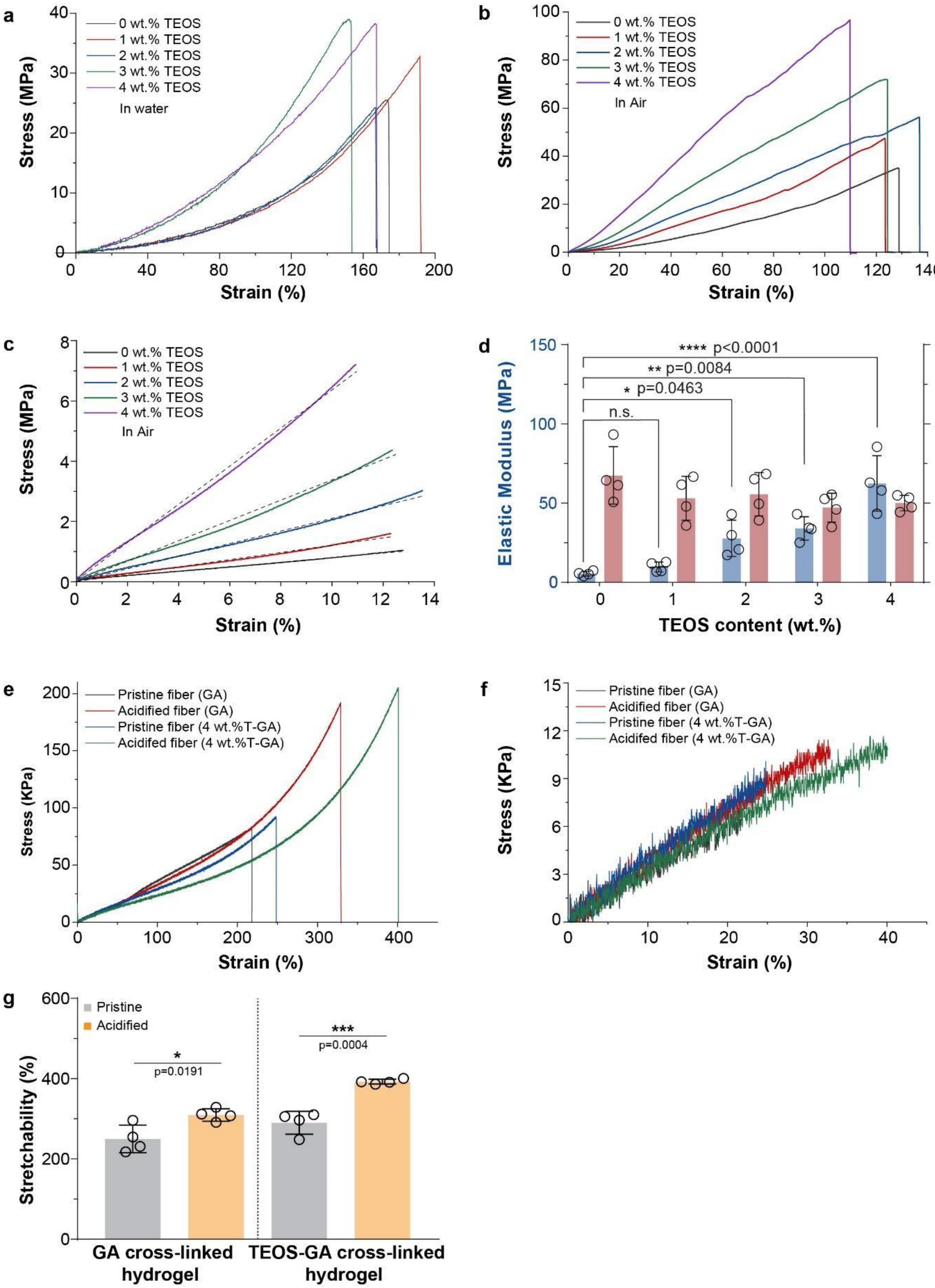

Supplementary Figure 16. Mechanical properties of COMPACT hydrogel fibers. a, Representative strass-strain curves of COMPACT hydrogel fibers with different TEOS

concentrations (0%-4%) tested in water. **b**, Representative strass-strain curves of COMPACT hydrogel fibers with different TEOS concentrations (0%-4%) tested in air with moisture. **c**, Zoomed-in elastic region (first 10% strain) of (**b)**. Dash lines represent slope fitting lines for elastic moduli extrapolation. **d**, Elastic modulus and stretchability (%) calculated from (b and c). (One-way ANOVA and Tukey's multiple comparisons tests were used for elastic modulus ($F_{4,15}$=20.51, ****p<0.0001) and stretchability ($F_{4,15}$=1.492, n.s. p=0.2543). Mean ± s.d., n=4 independent hydrogel fibers. **e**, Representative strass-strain curves of GA cross-linked hydrogel fiber and COMPACT hydrogel fiber (4 wt. % T). **f**, Zoomed-in elastic region (first 10% strain) of (**c**). **g**, Stretchability (%) of GA cross-linked hydrogel and TEOS-GA cross-linked hydrogels under pristine and acidified conditions, respectively. Acidified groups show significant differences (Two-tailed unpaired student's t-test, GA cross-linked hydrogel: F=4.887, t=3.178, df=6, *p=0.0191. TEOS-GA cross-linked hydrogels: F=22.05, t=7.005, df=6, ***p=0.0004). Mean ± s.d., n=4 independent hydrogel fibers.

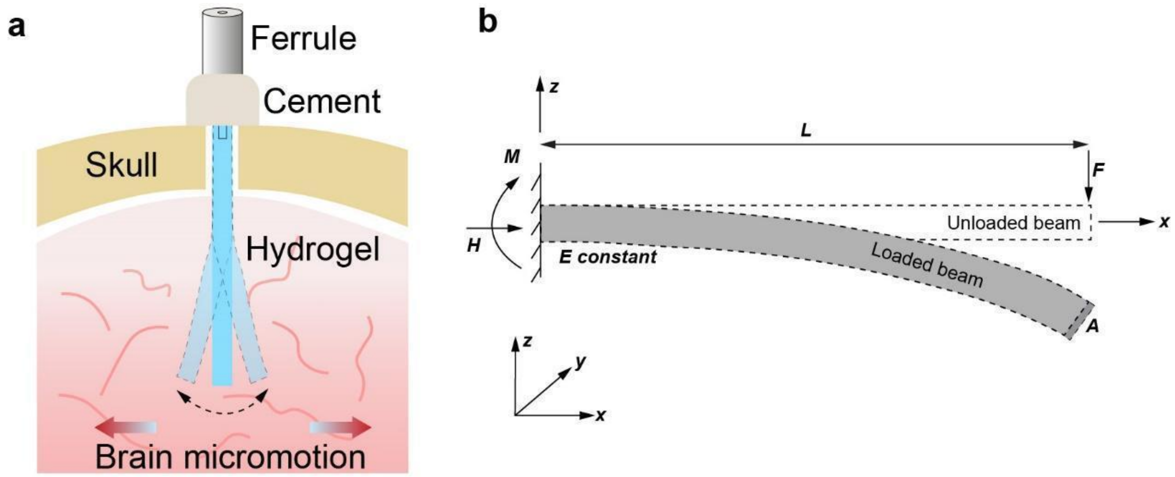

Supplementary Figure 17. Analysis of hydrogel fiber mechanics. a, An illustration of a hydrogel fiber inserted into a brain region, anchored at one end to the skull. This design allows the fiber to flexibly adapt to brain movements, facilitated by its low bending stiffness. **b,** A schematic of the cantilever beam bending model, which provides a framework for assessing the bending stiffness of the hydrogel fiber.

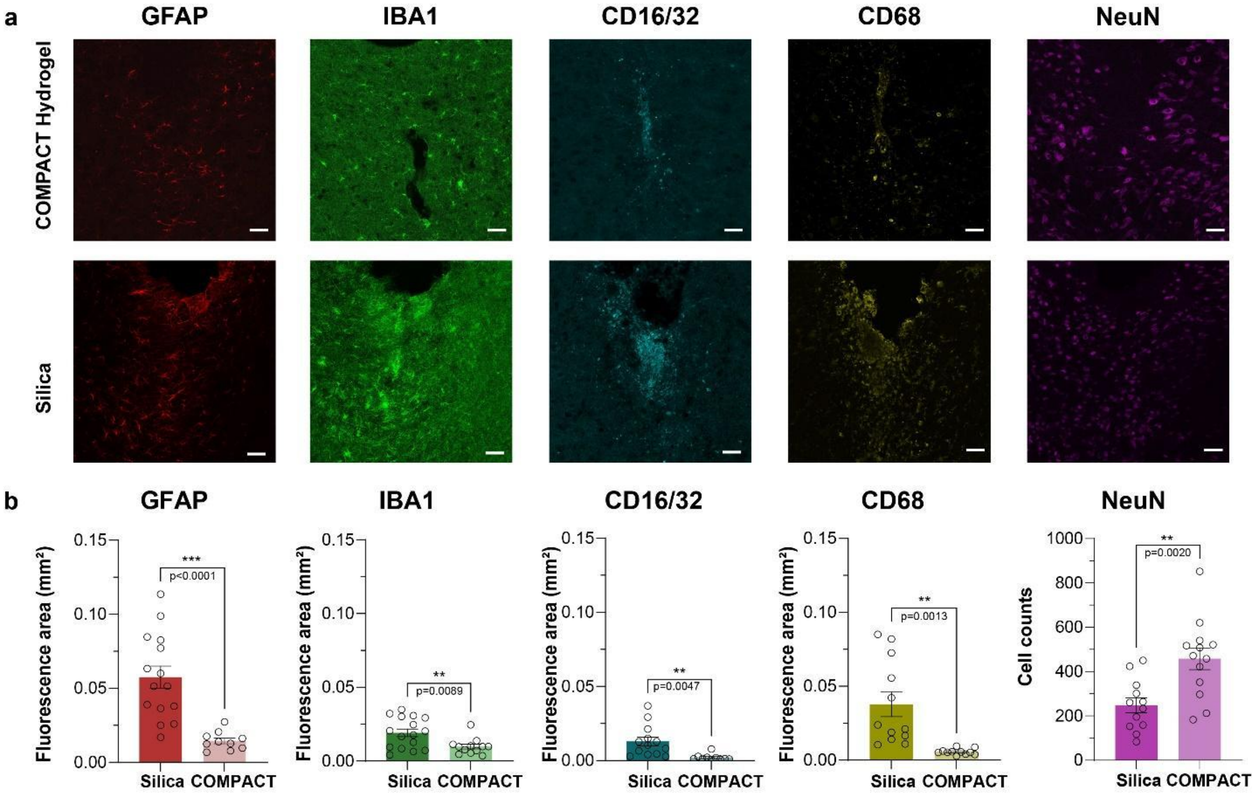

Supplementary Figure 18. Immunohistology of brain tissue reactions to implanted fibers 14 days post implantation. a, Representative confocal images of astrocytes (GFAP, red), microglia/macrophages (Iba1, green), presence of immunoglobulin G (CD16/32, cyan), activated macrophages (CD68, yellow), and neuronal cells (NeuN, magenta) surrounding a 400 μm hydrogel fiber probe (n=3 independent hydrogel fibers) and a 400 μm silica fiber (n=3 independent silica fibers) in mouse (n=3 mice) brain 14days after implantation. **b**, measured total area of antibodies labeled cells of astrocytes (GFAP, red), microglia/macrophages (Iba1, green), macrophages (CD16/32, cyan), macrophages (CD68, yellow), and neuronal cells (NeuN, magenta) surrounding a 400 μm hydrogel fiber probe (n=3) and a 400 μm silica fiber in mouse brain 14days after implantation. Two-tailed unpaired t-tests (α=0.05) were used to determine statistical significance. GFAP: F=21.44, t=4.638, df=23, ***p<0.0001. IBA1: F=3.475, t=2.829, df=26, **p=0.0089. CD16/32: F=25.12, t=3.519, df=21, **p=0.0047. CD68: F=210.3, t=3.697, df=21, **p=0.0013. NeuN: F=2.321, t=3.483, df=23, **p=0.0020. All the plots are presented as mean ± s.e.m., n=9-17 independent fluorescent images around the implantation sites. Confocal fluorescent imaging was repeated 10-30 times with similar results.

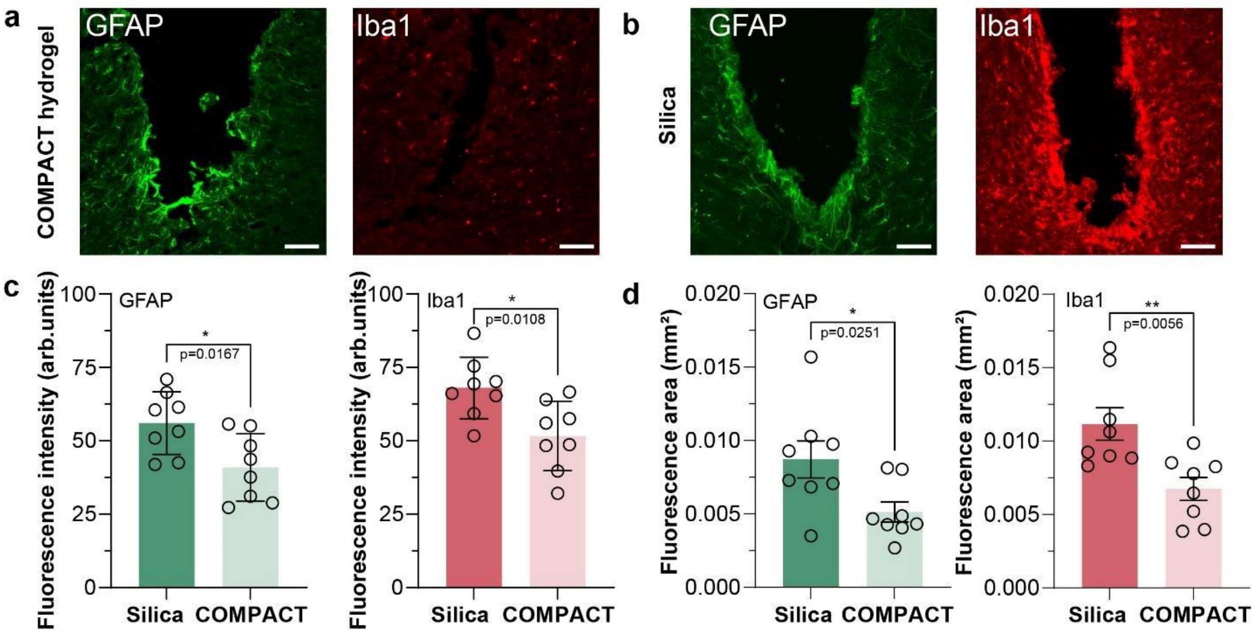

Supplementary Figure 19. Immunohistology of brain tissue reactions to implanted fibers one month post implantation. a, Representative confocal images of astrocytes (GFAP, green) and microglia (Iba1, red) surrounding a 400 μm hydrogel fiber probe (n=6 independent hydrogel fibers) in mouse (n=3 mice) brain 1 month after implantation. **b**, Representative optical images of astrocytes (GFAP, green) and microglia/macrophages (Iba1, red) surrounding a 400 μm silica fiber probe (n=6 independent silica fibers) in mouse (n=3 mice) brain 1 month after implantation. Scale: 100 μm. **c**, Left: counted GFAP fluorescence intensity from fibers implantation sites; Right: counted Iba1 fluorescence intensity from fibers implantation sites. (Two-tailed unpaired t-test (α=0.05), GFAP: F=1.157, t=2.717, df=14, *p=0.0167. Iba1: F=1.280, t=2.937, df=14, *p=0.0108). **d,** Left: counted total area of astrocytes around fibers implantation sites; Right: Left: counted total area of microglia around fibers implantation sites. (Two-tailed unpaired t-test (α=0.05), GFAP: F=3.341, t=2.508, df=14, *p=0.0251. Iba1: F=1.959, t=3.269, df=14, *p=0.0056). All the plots are presented as mean ± s.e.m., n=8 independent fluorescent images around the implantation sites. Confocal fluorescent imaging was repeated 10-30 times with similar results.

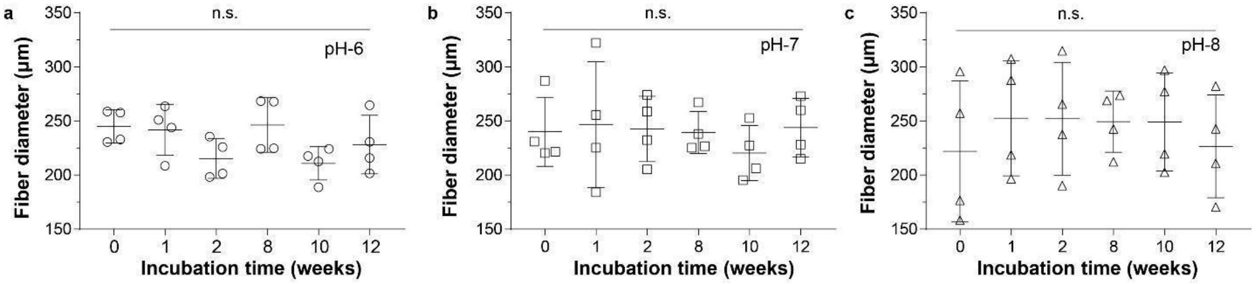

Supplementary Figure 20. Stability of COMPACT hydrogel fibers. a, Diameters of COMPACT hydrogel fibers (4% TEOS, 800 μm mold) in saline solutions (pH=6) at 37 ^{o}C over 12 weeks (One-way ANOVA and Tukey's multiple comparisons test, $F_{5, 18}$=2.124, n.s. p=0.1093). **b**, Diameters of COMPACT hydrogel fibers (4% TEOS, 800 μm mold) in saline solutions (pH=7) at 37 ^{o}C over 12 weeks (One-way ANOVA and Tukey's multiple comparisons test, $F_{5, 18}$=0.3029, n.s. p=0.9048). **c**, Diameters of COMPACT hydrogel fibers (4% TEOS, 800 μm mold) in saline solutions (pH=8) at 37 ^{o}C over 12 weeks (One-way ANOVA and Tukey's multiple comparisons test, $F_{5, 18}$=0.3089, n.s. p=0.9011). All the plots are presented as mean ± s.d., n=4 independent hydrogel fibers.

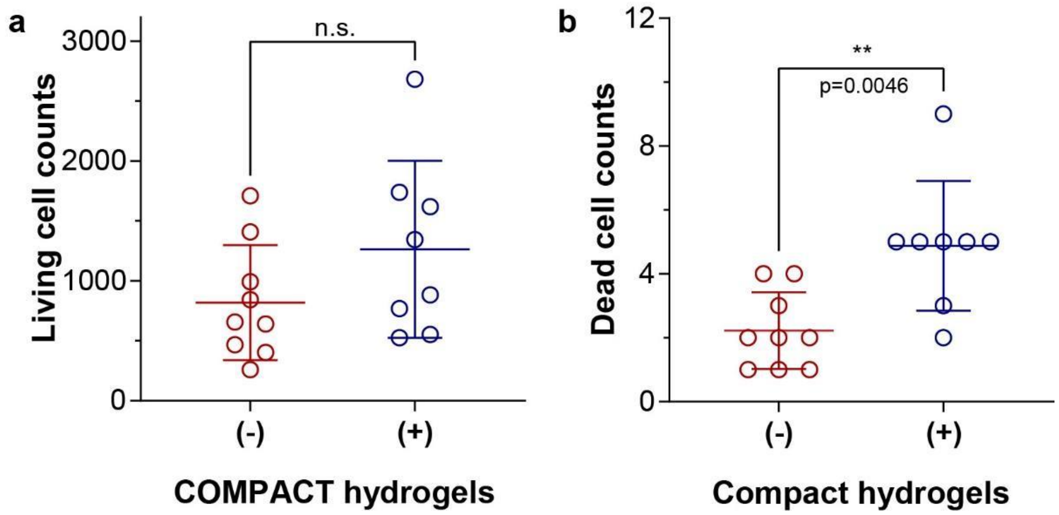

Supplementary Figure 21. Cytotoxicity of COMPACT hydrogel fibers. a, Living cell measurements of groups with hydrogel incubations and without hydrogel incubations. Living cell measurements show no significant difference between groups (Two-tailed unpaired t-test (α=0.05): F=2.372, t=1.490, df=15, n.s. p=0.1569). **b**, Dead cell measurements of groups with hydrogel incubations and without hydrogel incubations. Deal cell counts show no significant difference between groups (Two-tailed unpaired t-test (α=0.05): F=2.856, t=3.325, df=15, **p=0.0046). All the plots are presented as mean ± s.e.m., n=8-9 independent fluorescent images.

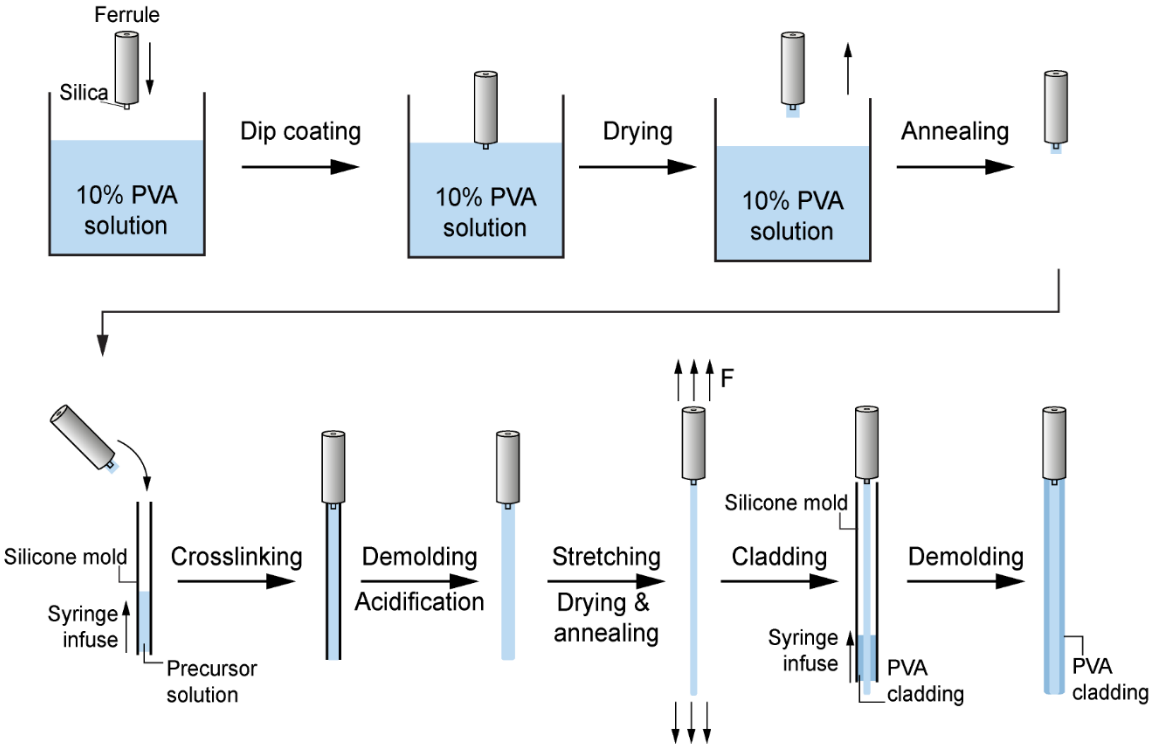

Supplementary Figure 22. Step-index hydrogel fiber optic device fabrication with core-cladding structures. A 13 mm-long silica segment with a diameter of 400 µm was integrated with an optical ferrule with a 400 µm bore via optical glue. Both ends of the silica segment were polished to ensure optimal light transmission. The extruded part of the silica segment from the ferrule was washed with 75% ethanol and treated with plasma to enhance the hydrophilicity of its surface. A thin layer of 10 wt.% PVA was dip-coated on the extruded silica fiber and dried at RT for 12 hours, followed by annealing at 100 °C for 2 hours. To produce COMPACT optical fiber, the precursor solution (**Supplementary Figure 1**) was infused into a silicone mold with a diameter of 800 µm, and the PVA-coated silica-ferrule was inserted into the other end of the mold. The PVA hydrogel was allowed to cross-link and connect with the extruded silica segment at RT for 4 hours. After demolding, acidification (12 mM HCl, 2 hours) was introduced. The ferrule-connected fiber was stretched and dried at 60 °C for 12 hours, with additional annealing at 100 °C for 20 minutes. To produce a COMPACT optical device with core-cladding structures, the annealed ferrule-connected fiber was re-inserted into a silicone mold with a diameter of 500 µm. Glutaraldehyde (GA) was added to a 5 wt.% PVA solution and processed by degassing and mixing. HCl was added to a 5 wt.% PVA solution, followed by degassing and mixing. The above two solutions were mixed (weight ratio of 1: 1) and degassed. The mixture of PVA-GA-HCl was infused into the 500 µm silicone mold to cross-link (RT, 4 hours) and form a cladding layer around the optical fiber. The COMPACT optical device with core-cladding structures was obtained after demolding. The mixing parameters of the planetary mixer were consistent: 2000 rpm, 1 minute, and a vacuum of 16 kPa.

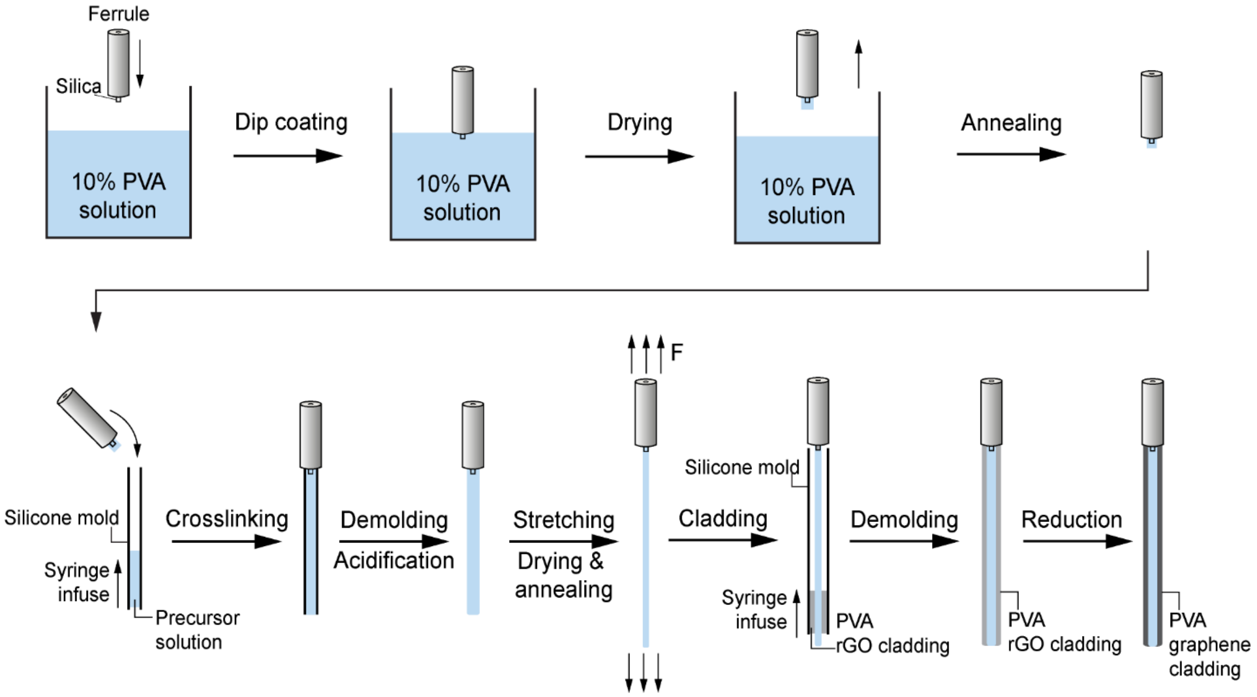

Supplementary Figure 23. Step-index hydrogel fiber optic device fabrication with reduced graphene oxide (rGO) claddings. To fabricate a compact optical device with reduced graphene oxide (rGO) claddings, we utilized the same approaches as those used for assembling silica segments and ferrules to fabricate compact optical fibers (as described in **Supplementary Figure 22**). Firstly, the annealed ferrule-connected fiber was re-inserted into a silicone mold with a diameter of 500 µm. Next, we added GA into a 5 wt.% PVA solution and degassed the mixture. Similarly, hydrochloric acid (HCl) was added into another 5 wt.% PVA solution and degassed. The two solutions were then mixed with a graphene oxide solution (weight ratio of 1:1:1) and degassed. The resulting mixture of PVA-GA-HCl-rGO was infused into the 500 µm silicone mold and allowed to cross-link at room temperature for 4 hours, thereby forming a cladding layer around the optical fiber. To elute the core-cladding optical device, we used dichloromethane (DCM). Subsequently, we incubated the compact optical device with the core-cladding in a 20 mL aqueous solution containing 1 g of sodium hydroxide and 0.3 g of sodium hydrosulfite at 40 °C for 1 minute to achieve a graphene cladding. Throughout the process, we maintained consistent mixing parameters using a planetary mixer at 2000 rpm for 1 minute under a vacuum of 16 kPa.

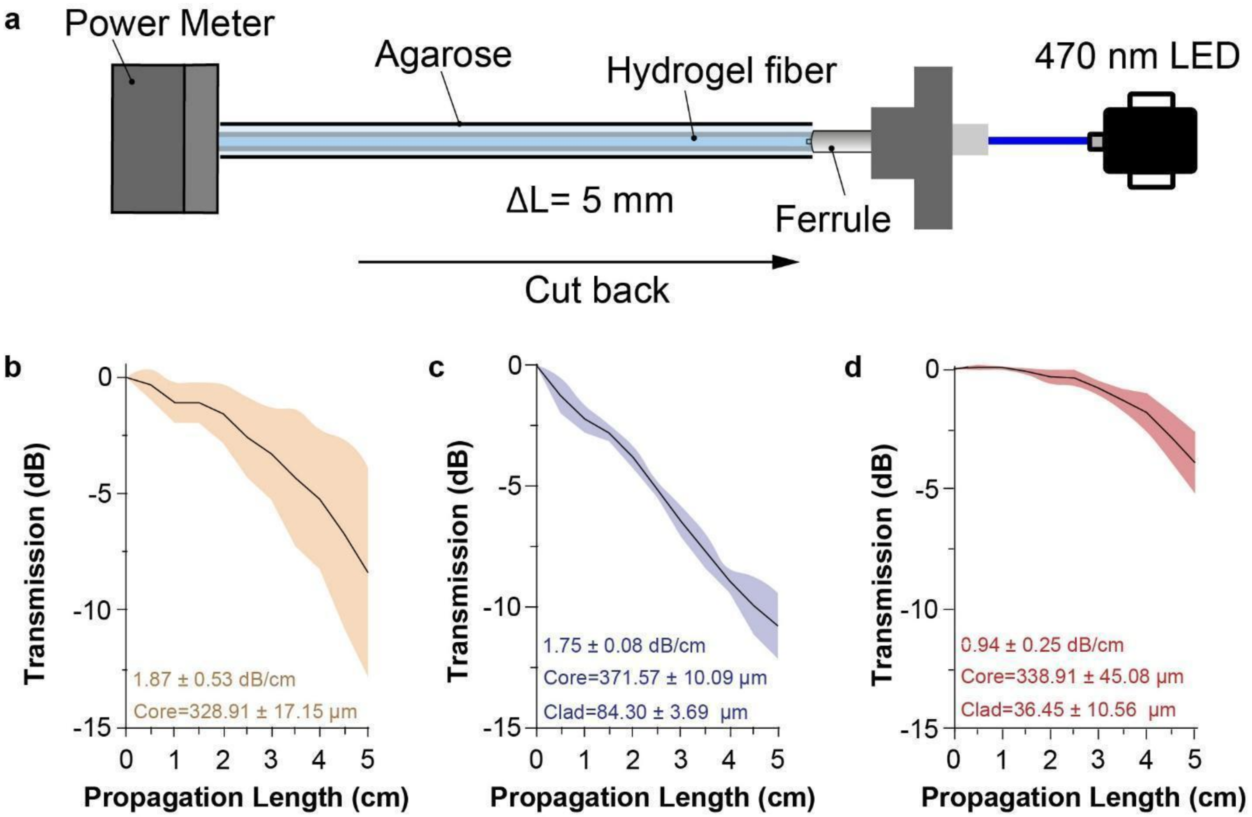

Supplementary Figure 24. Characterization of light transmission of COMPACT hydrogel fibers. a, A schematic of the cut-back method for light attenuation characterization (λ=470 nm). Hydrogel fibers were connected with a silica segment (embedded in an optical ferrule) and encapsulated by 1% agarose to maintain hydration. After each cut (increment 5 mm), transmitted power through hydrogel fibers was measured. LED: laser-emitting diode. **b**, Normalized transmission (λ=470 nm) as a function of fiber length with core-only hydrogel fibers (3% TEOS, n=4 independent optical probes, mean ± s.d.). **c**, Normalized transmission (λ=470 nm) as a function of fiber length with core-cladding hydrogel fibers (3% TEOS, 5% PVA cladding, n=4 independent optical probes, mean ± s.d.). **d**, Normalized transmission (λ=470 nm) as a function of fiber length with core-cladding hydrogel fibers (3% TEOS, 5% PVA-graphene cladding, n=4 independent optical probes, mean ± s.d.).

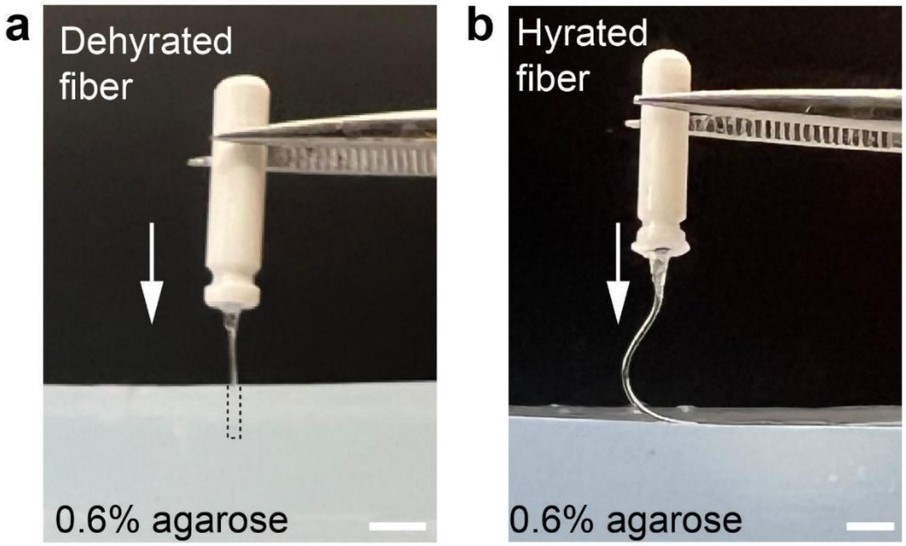

Supplementary Figure 25. Implantation test COMPACT hydrogel fibers. a, Insertion of an optical fiber probe under dehydrated status into the phantom brain (0.6% agarose). Scale: 2.5 mm. **b**, Buckling of an optical fiber probe under hydrated status during the insertion into the phantom brain (0.6% agarose). Scale: 2.5 mm.

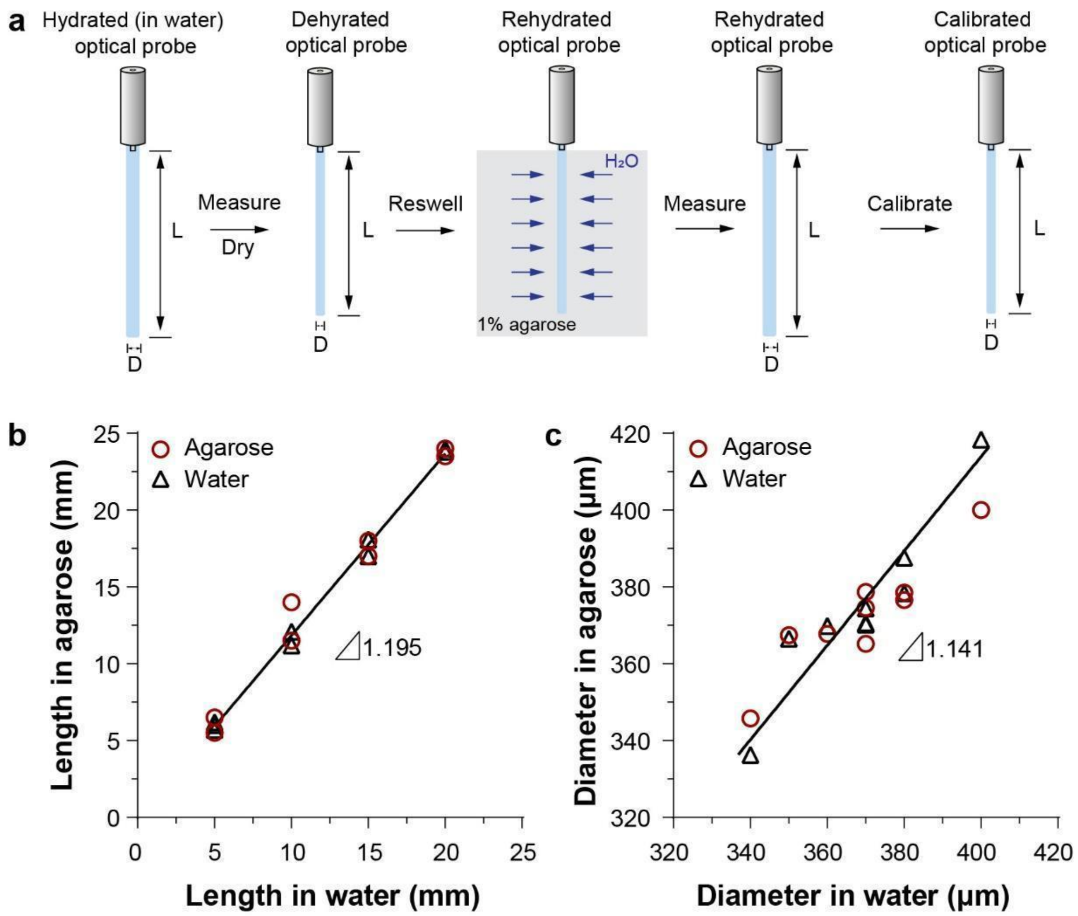

Supplementary Figure 26. Calibration of COMPACT hydrogel fibers for implantations. a, Process for calibrating swelling ratio from dehydrated to hydrated status with water and 1% agarose. **b**, Length calibration (relation between 1% agarose and water, fitting slope 1.195) of optical fibers. Each dot indicates one independent optical probe. **c**, Diameter calibration (relation between 1% agarose and water, fitting slope 1.141) of optical fibers. Each dot indicates one independent optical probe.

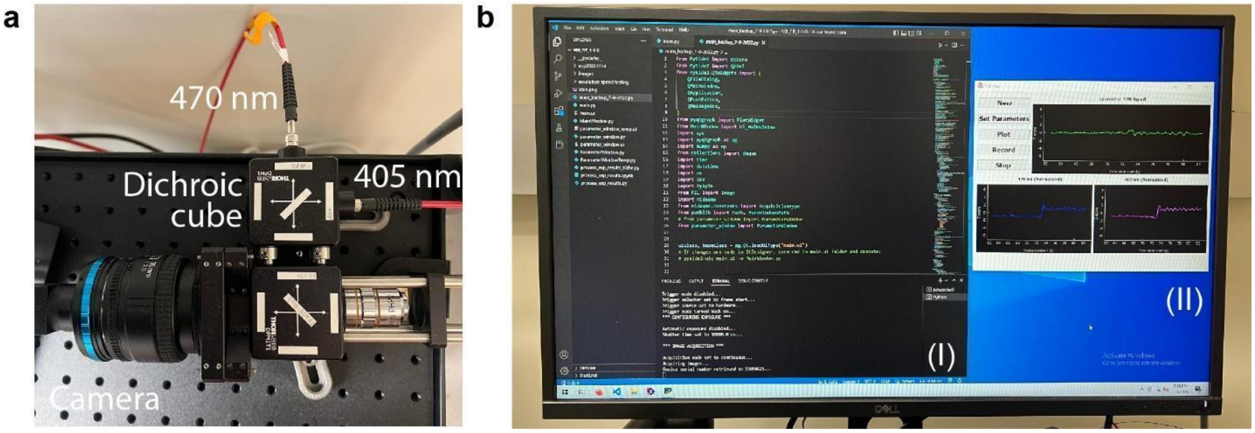

Supplementary Figure 27. Fiber photometry apparatus a, A photograph of the fiber photometry system setup consisting of optical components: 470 nm LED, 405 nm LED, dichroic mirrors, and camera. **b**, (I) a customized python coding for LED control and images acquisition, (II) a real-time monitoring window of GCaMP fluorescence signals during social interactions.

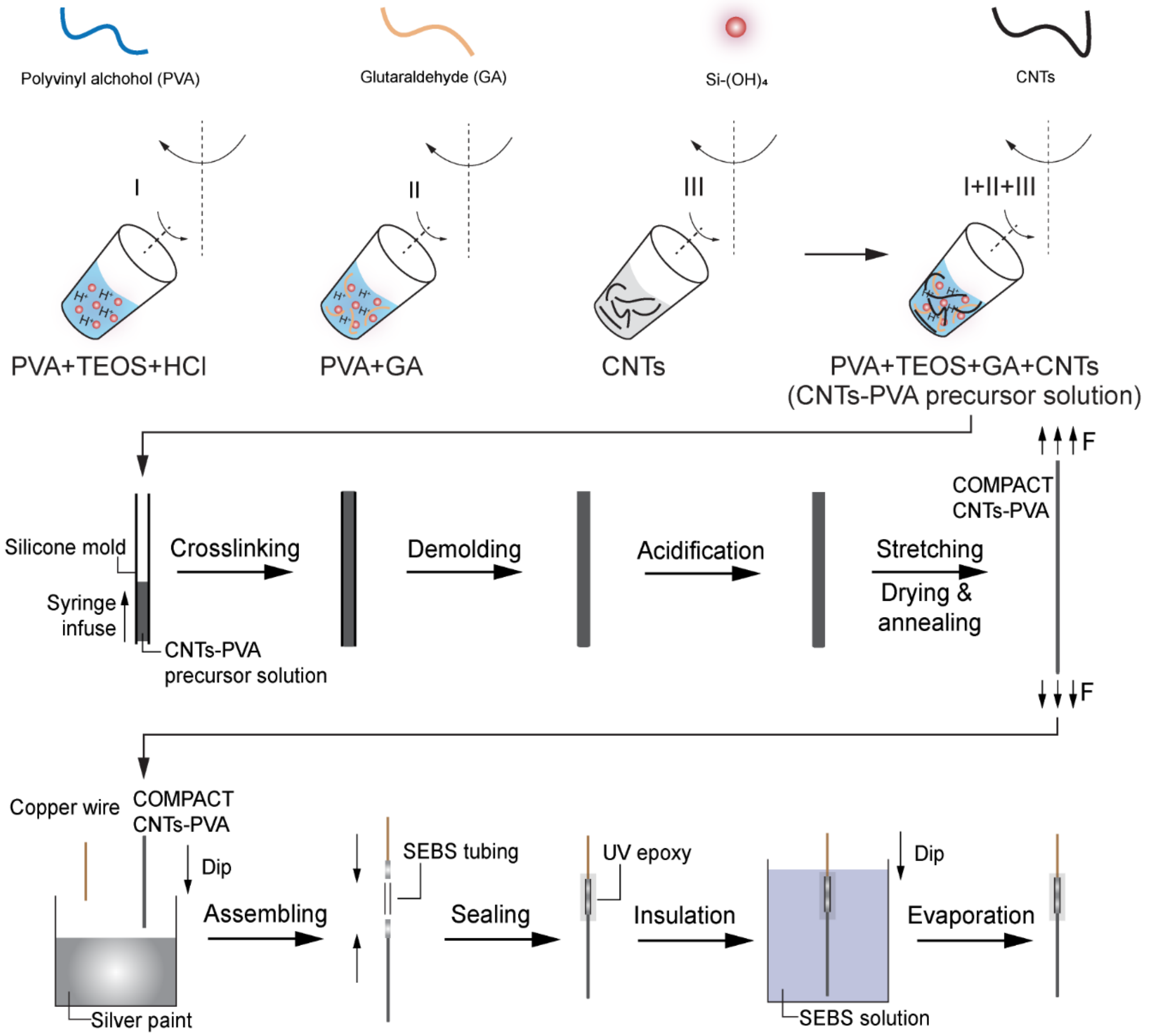

Supplementary Figure 28. Carbon-nanotubes (CNTs) loaded PVA hydrogel electrode fabrication. To incorporate CNTs (length to diameter ratio 2000-10000:1, TUBALL PD0634) into the PVA matrix, the same methods of TEOS hydrolysis (**Supplementary Figure 1**) and mixing strategy were used. The CNTs solution was dispersed in Sodium dodecylbenzene sulfonate (SDBS) to prevent aggregation. The dispersed CNTs solution was mixed with PVA-TEOS-HCl solution and PVA-GA solution (weight ratio=0.4:1:1) and degassed. To fabricate CNTs-VA hydrogel fibers, the same procedure of fiber molding and extrusion was adapted (**Supplementary Figure 2).** To fabricate CNTs-PVA hydrogel electrodes, a CNTs-PVA hydrogel fiber and a copper wire were dip-coated in silver paint. The dip-coated hydrogel fiber and copper wire were inserted into elastic tubing (100 µm) to create a hydrogel-copper junction. Another thin layer of silver was applied at the junction to enhance conductivity. UV epoxy was used to seal and reinforce the junction. To insulate the CNTs-PVA hydrogel fiber, a SEBS solution (20 wt.% SEBS in toluene) was used for dip coating. The CNTs-PVA hydrogel electrode was acquired after the evaporation of SEBS solutions.

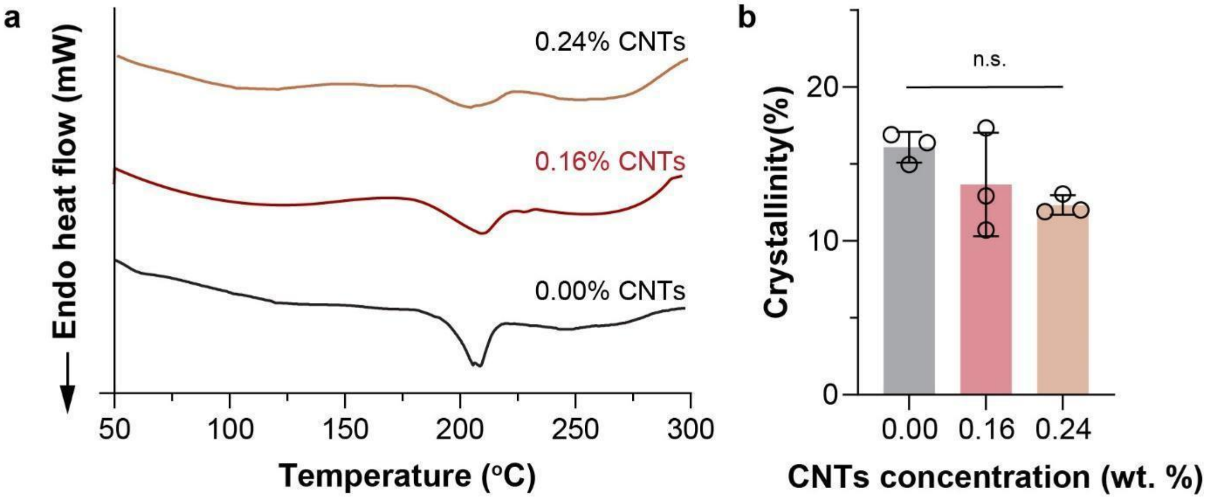

Supplementary Figure 29. **Characterization of crystallinity in CNTs-PVA hydrogels. a,** Representative DSC profiles of CNTs-PVA hydrogel fibers including different CNTs contents (0.00%, 0.16% and 0.24%, with acidification and 200% stretching). **b,** Crystallinity (%) of COMPACT hydrogel fibers calculated from a (One-way ANOVA and Tukey's multiple comparisons tests, $F_{2,6}$=2.565, n.s. p=0.1567). Mean ± s.d., n=3 independent hydrogel fibers.

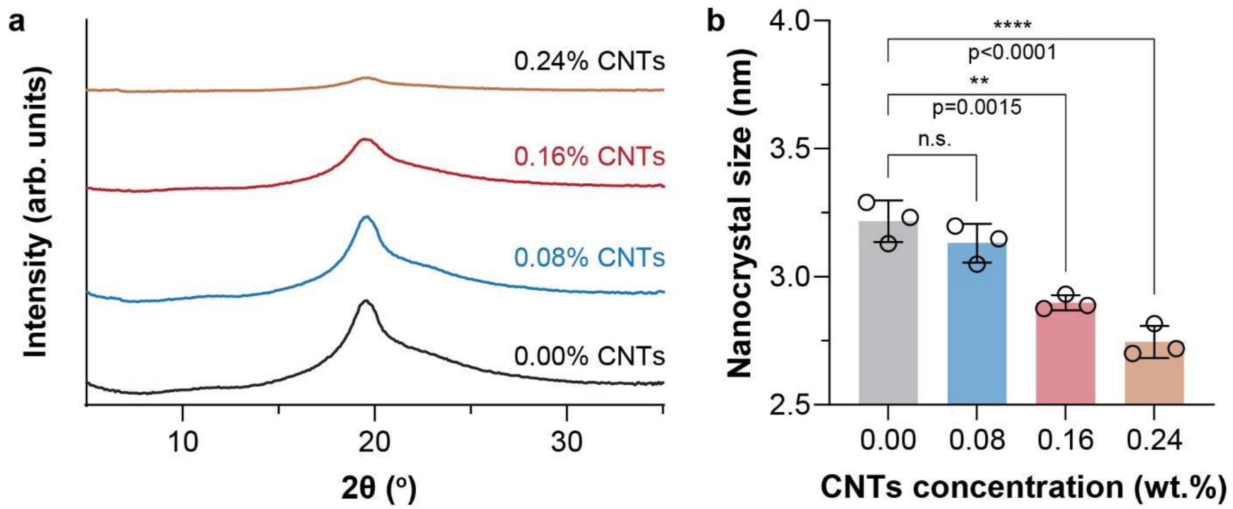

Supplementary Figure 30. **Characterization of nanocrystal size in CNTs-PVA hydrogels. a,** Representative WAXS curves of CNTs-PVA hydrogel fibers including different CNTs contents (0.00%, 0.08%, 0.16% and 0.24%, with acidification and 200% stretching). **b,** Nanocrystal size of CNTs-PVA hydrogel fibers calculated from a (One-way ANOVA and Tukey's multiple comparisons tests, $F_{3, 8}$=32.56, ****p<0.0001). Mean ± s.d., n=3 independent hydrogel fibers.

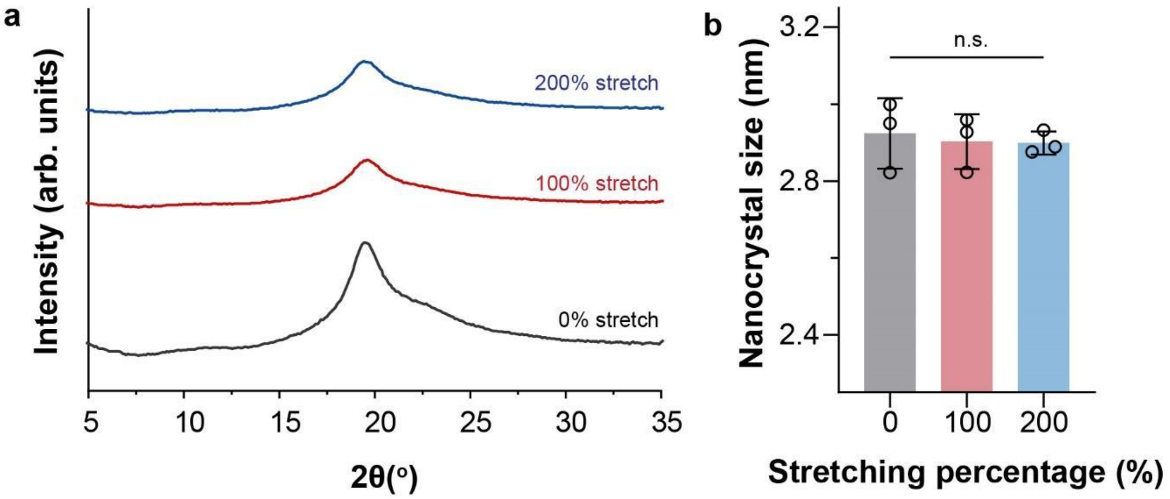

Supplementary Figure 31. The effect of stretching on nanocrystal size in CNTs-PVA hydrogel fibers. a, Representative WAXS profiles collected from CNTs-PVA hydrogel fibers (0.16%CNTs, with acidification) under different stretching percentages (0%, 100% and 200%)**. b,** Nanocrystal size of CNTs-PVA hydrogel fibers calculated from **a** (One-way ANOVA and Tukey's multiple comparisons tests, $F_{2, 6}$=0.1113, n.s. p=0.8965). Mean ± s.d., n=3 independent hydrogel fibers.

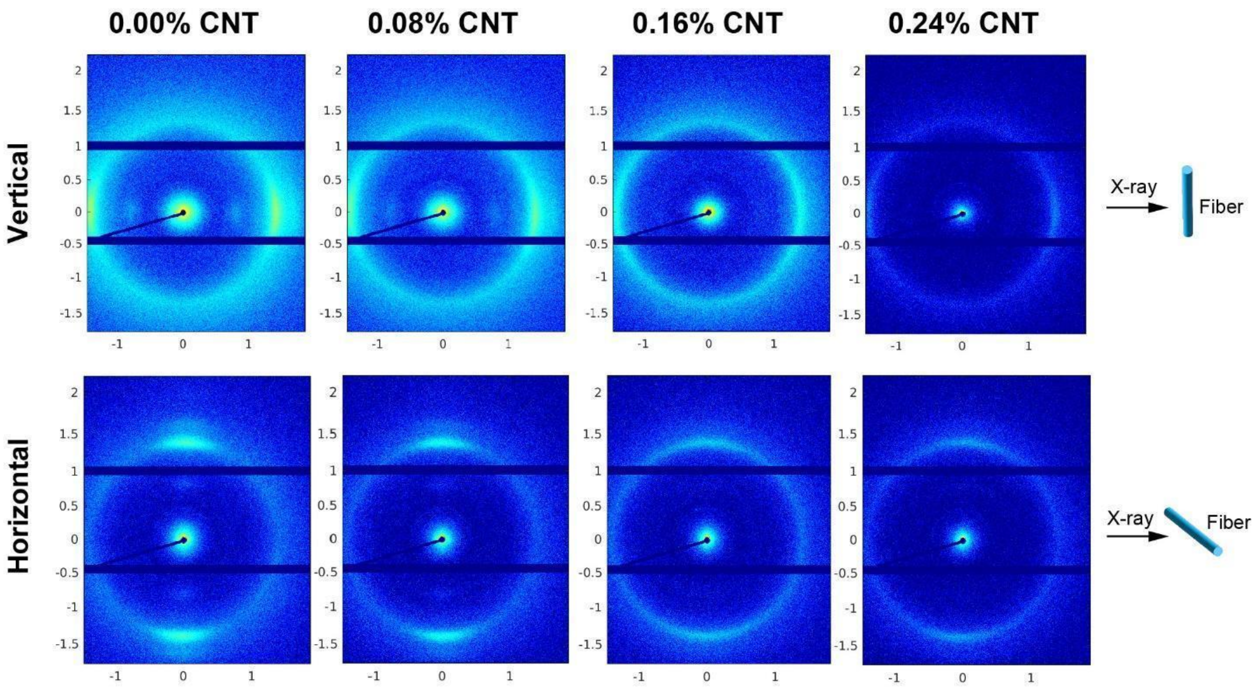

Supplementary Figure 32. **WAXS 2D patterns of nanocrystalline domain orientations in CNTs-PVA hydrogels with different CNTs concentrations.** Representative WAXS 2D patterns (vertical and horizontal measurements) of COMPACT hydrogel fibers including different CNTs concentrations (0.00%, 0.08%, 0.16%, and 0.24%, with acidification and 200% stretching).

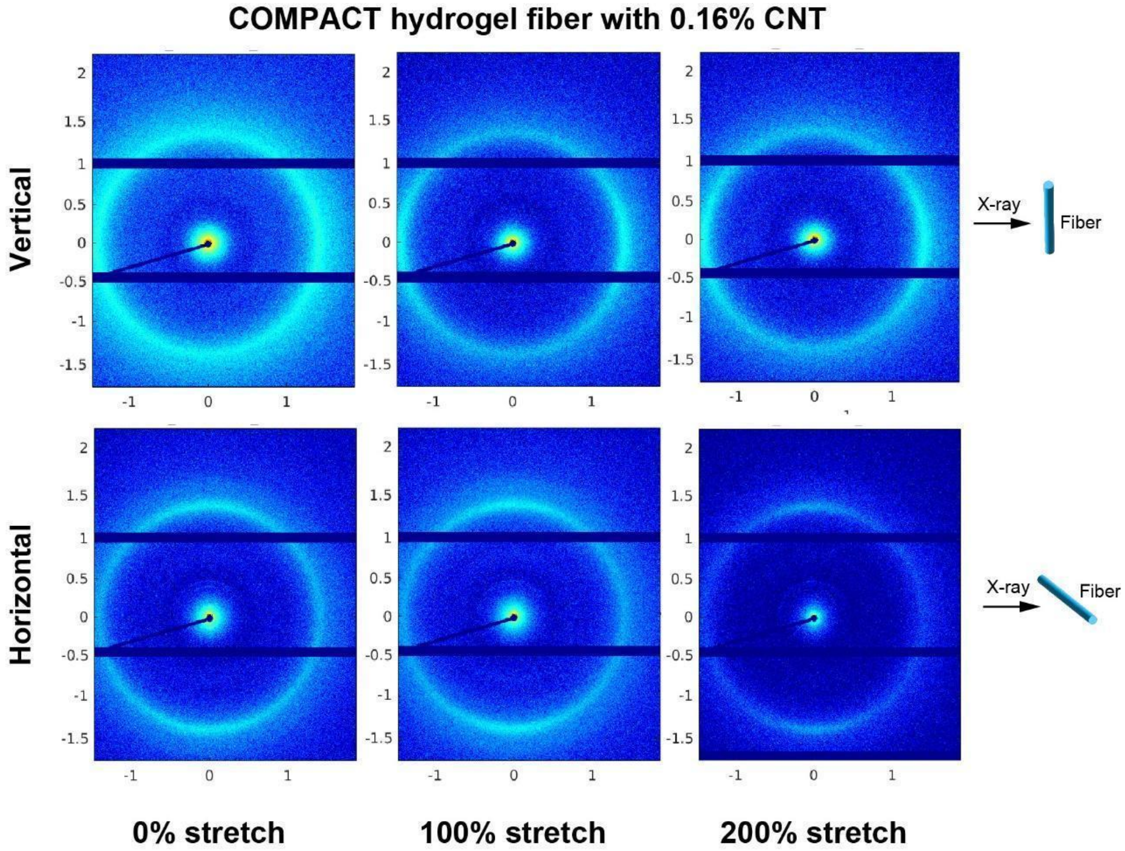

Supplementary Figure 33. WAXS 2D patterns of nanocrystalline domain orientations in CNTs-PVA hydrogels under different stretching percentages. Representative WAXS 2D patterns collected from CNTs-PVA hydrogel fibers (0.16%CNTs, with acidification) under different stretching percentages (0%, 100% and 200%).

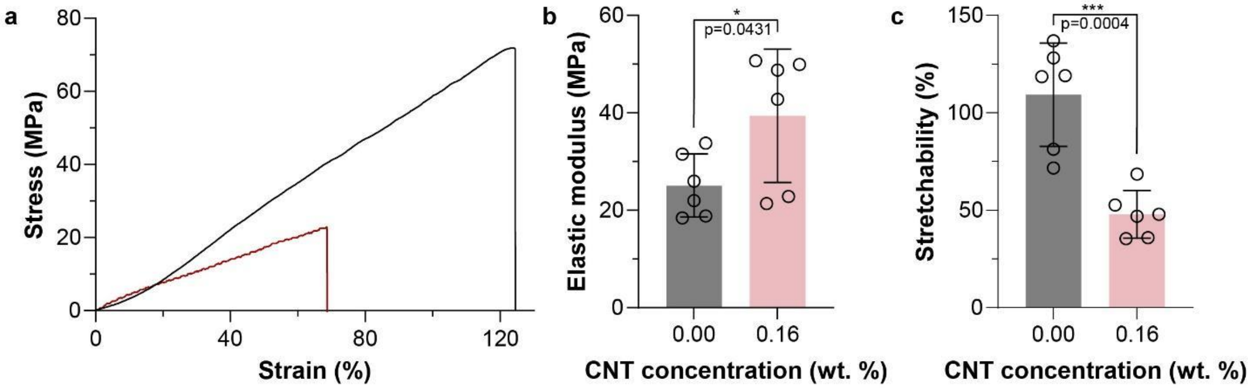

Supplementary Figure 34. Mechanical properties of CNTs-PVA hydrogel fibers. a, Representative stress-strain curves of CNTs-PVA hydrogel fibers (0.00 wt.% CNTs and 0.16 wt.% CNTs). **b,** Elastic modulus comparisons of CNTs-PVA hydrogel fibers' (0.00 wt.% CNTs and 0.16 wt.% CNTs). **c**, Stretchability (%) comparisons of CNTs-PVA hydrogel fibers' (0.00 wt.% CNTs and 0.16 wt.% CNTs). Two-tailed unpaired t-tests (α=0.05) were used to determine the statistical significance (Elastic modulus: F=4.460, t=2.316, df=10, *p=0.0431. Stretchability: F=4.677, t=5.157, df=10, ***p=0.0004). All the plots (**b** and **c**) are presented as mean ± s.d., n=6 independent hydrogel fibers.

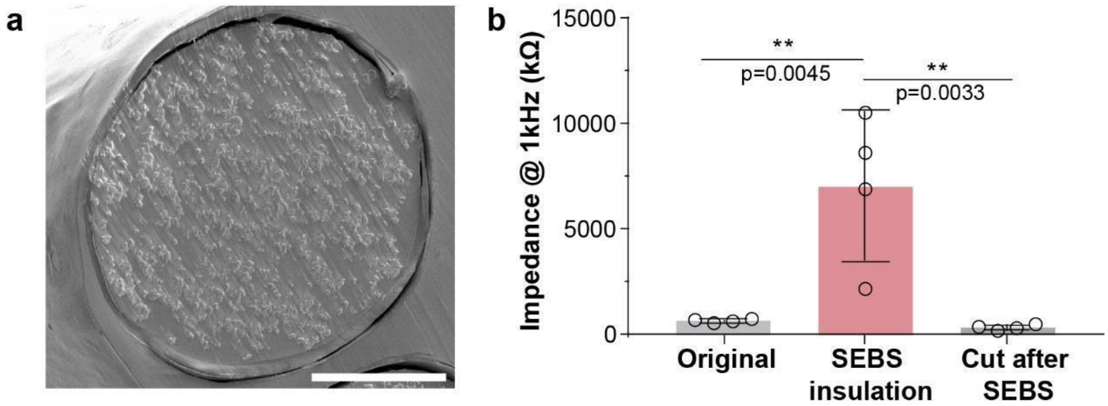

Supplementary Figure 35. Insulation of COMPACT hydrogel electrodes. a, An SEM image of a CNTs-PVA hydrogel electrode insulated with SEBS (thickness:4.55 ± 1.32 µm). Scale: 50 µm. **b**, Impedance at 1 kHz of hydrogel electrodes under pristine state, with SEBS insulation, and tip exposure after SEBS insulation. SEBS-insulated electrodes show a significant difference as compared to the other two groups (One-way ANOVA and Tukey's multiple comparisons test, $F_{2,9}$=13.43, **p=0.0020). Mean ± s.d., n=4 independent hydrogel microelectrodes.

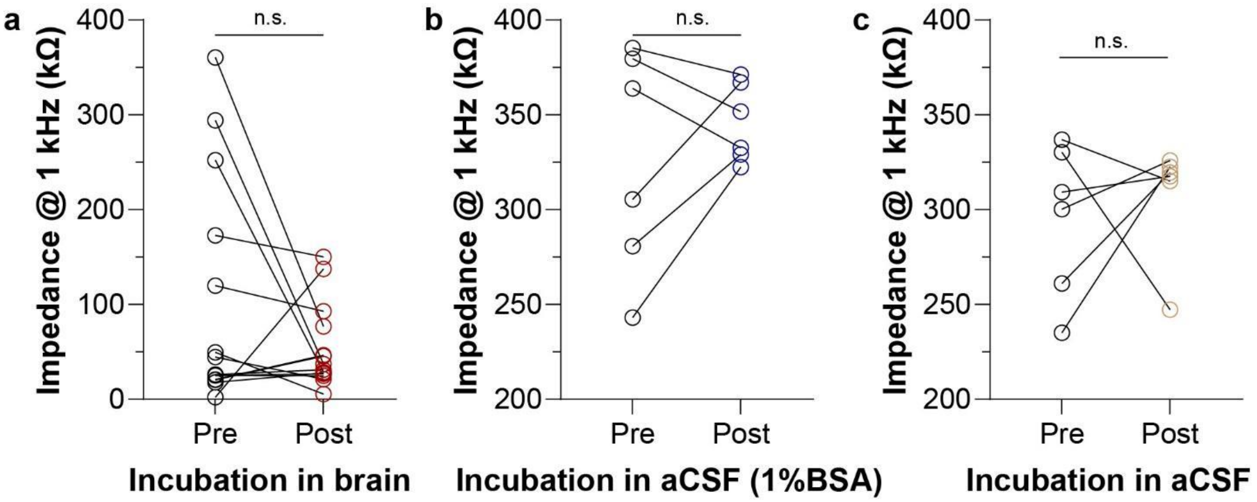

Supplementary Figure 36. In vivo and in vitro incubation of COMPACT hydrogel electrodes. **a**, Impedance at 1 kHz of COMPACT hydrogel microelectrodes (3% TEOS with 0.16% CNT) before and after 14 days incubation in brains of mice (n=2 mice). **b**, Impedance at 1 kHz of COMPACT hydrogel microelectrodes (3% TEOS with 0.16% CNT) before and after 14 days incubation (37 °C) artificial cerebrospinal fluid (aCSF). **c**, Impedance at 1 kHz of COMPACT hydrogel microelectrodes (3% TEOS with 0.16% CNT) before and after 14 days incubation (37 °C) aCSF with 1% bovine serum albumin (BSA). Two-tailed paired student t-tests (α=0.05) were used to determine the statistical significance. Incubation in brain: t=1.512, df=13, n.s. p=0.1544. Incubation in aCSF (1% BSA): t=0.9635, df=5, n.s. p=0.3796. Incubation in aCSF: t=0.5095, df=5, n.s. p=0.6321. Each individual dot presents one individual hydro microelectrode (n=6-14 independent hydrogel microelectrodes).

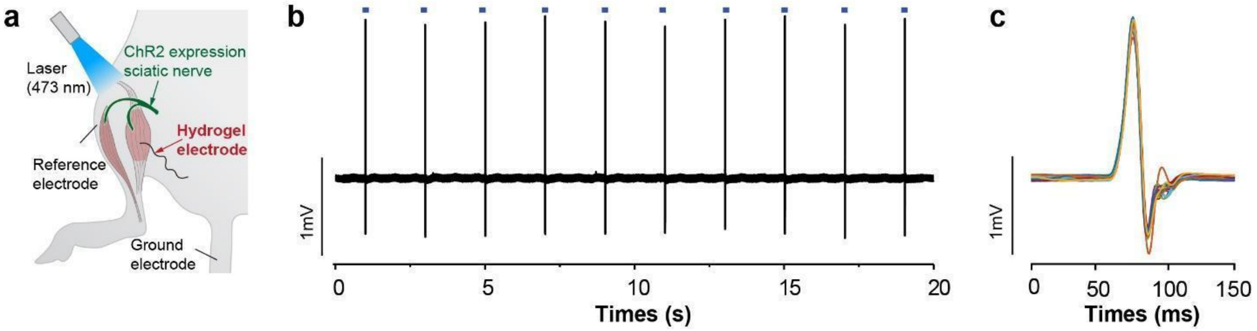

Supplementary Figure 37. Electromyographic (EMG) recordings of mouse hindlimb muscles with CNTs-PVA hydrogel electrodes. a, A schematic illustration of electrical recordings from mouse gastrocnemius muscles with a CNTs-PVA electrode in the presence of transdermal optical stimulation. h, Representative EMG signals recorded with CNTs-PVA hydrogel electrodes upon transdermal optogenetic stimulations in *Thy1::ChR2-EYFP* mice (λ=473 nm, 0.5 Hz, pulse width 50 ms, 200 mW/mm^2). Blue bars indicate the light illumination periods. i, Overlay plot of EMG peaks.

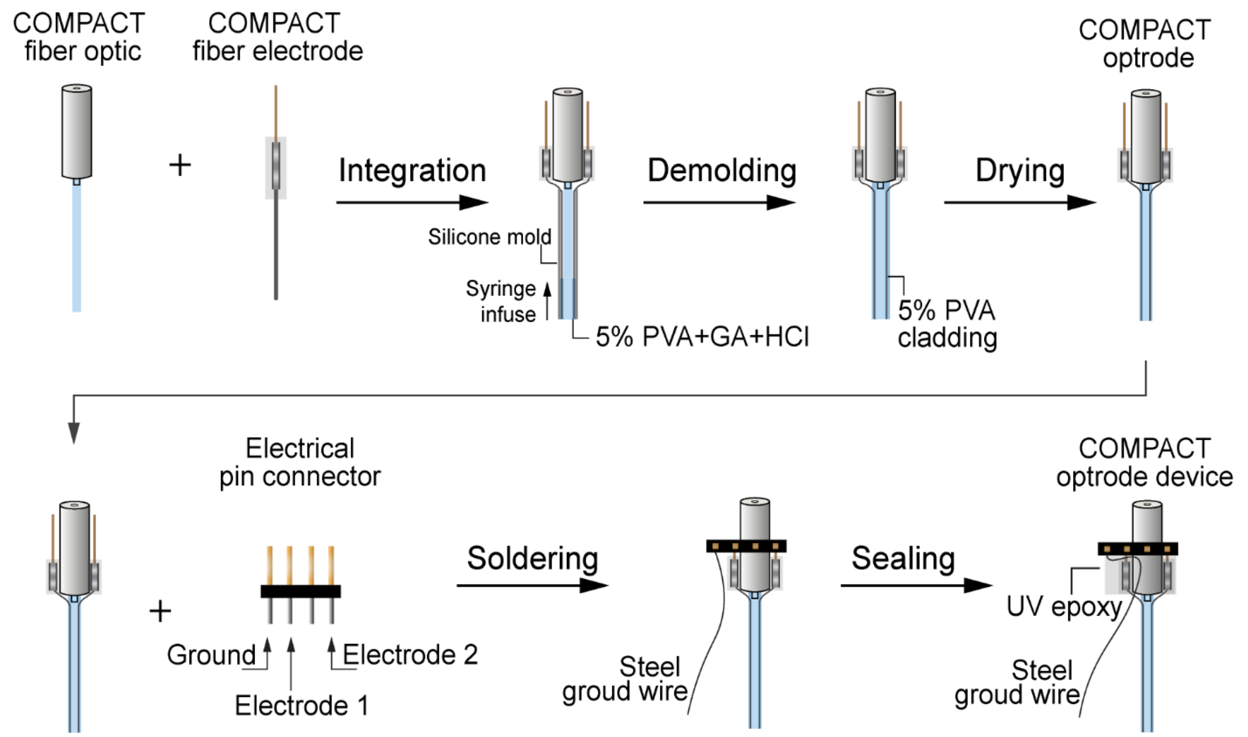

Supplementary Figure 38. Hydrogel optrode fabrication. To fabricate COMPACT optrode fibers, a COMPACT fiber optic, and 2 COMPACT fiber electrodes were inserted and aligned in a silicone mold (500 µm). GA was added into a 5 wt.% PVA solution and processed by degassing and mixing. HCl was added into a 5 wt.% PVA solution with degassing and mixing. The above two solutions were mixed (weight ratio=1:1) and degassed. The mixture of PVA-GA-HCl was injected into the silicone mold to cross-link (RT, 4 hours) and form a cladding layer around the optical fiber and electrodes. To fabricate COMPACT optrode devices, a 4-pin electrical pin connector was used to connect 2 electrodes, and a steel wire (50 µm diameter) was used for grounding. UV epoxy was used to seal and reinforce the soldering junction between the electrical pin connector and electrodes.

Supplementary Table 1. Crystallinity% calculation from DSC results in Fig.7j.

	ΔH_m (mJ/mg)	ΔH_f (mJ/mg)	Cry%=$\Delta H_m/\Delta H_{100}$ ·100 (%)
PVA	40.9 ± 20.6	150	28.4 ± 3.5
COMPACT (-)	32.4 ± 1.6	150	21.6 ± 1.1
COMPACT (+)	19.1 ± 2.2	150	12.7 ± 1.5

Supplementary Table 2. Crystallinity% calculation from DSC results in SI Fig. 8b.

	ΔH_m (mJ/mg)	ΔH_f (mJ/mg)	Cry%=$\Delta H_m/\Delta H_{100}$ ·100 (%)
0% TEOS	32.4 ± 1.6	150	21.6 ± 1.1
3% TEOS	24.1 ± 1.5	150	16.1 ± 1.0
4% TEOS	19.1 ± 2.2	150	12.7 ± 1.5

Supplementary Table 3. Information of SAXS data collection.

Source, instrument and description or reference	Cu Ka stationary source, Ganesha SAXSLAB 300XLwith a Dectris Pilatus 300K 2D CMOS photon counting detector (size: 83.8 × 106.5 mm^2)
Wavelength (Å)	1.54
Beam geometry (size, sample-to-detector distance)	Square (0.9 x 0.9 mm), 100.4 mm
q-measurement range ($Å^{-1}$ or nm^{-1})	0.0025 to 0.3000
Absolute scaling method	The absolute scattering intensity is measured by normalizing the measured intensity with the direct incident beam intensity.
Basis for normalization to constant counts	The incident beam intensity is measured with the same 2D detector by removing the beam stop. Linearity of detector is confirmed.
Method for monitoring radiation damage, X-ray dose where relevant	Not relevant in this study
Exposure time, number of exposures	600s, 3 times
Sample configuration including path length and flow rate where relevant	Not relevant in this study
Sample temperature (ºC)	25

Supplementary Table 4. Crystallinity% calculation from DSC results in SI Fig. 29b.

	ΔH_m (mJ/mg)	ΔH_f (mJ/mg)	Cry%=$\Delta H_m/\Delta H_{100} \cdot 100$
0.00%CNTs	24.1± 1.5	150	16.1 ± 1.0
0.16%CNTs	20.5 ± 5.1	150	13.7 ± 3.4
0.24%CNTs	18.5± 1.0	150	12.3 ± 0.6

The degree of crystallinity α can be estimated from the formula: $\alpha = 100\% \cdot \Delta H_m / \Delta H_f$[1]

α: crystallinity (%)

ΔH_m: experimental melting enthalpy of crystallinity in PVA measured by integrating endothermic peaks of PVA from thermographs.

ΔH_f: enthalpy of melting 100% crystallinity in PVA[2, 3].

Supplementary Table 5. Mouse information for every in vivo experiment.

Mouse ID	Strain	Sex	Age at surgery	Experiments	Notes
C1M1	C57BL/6	Female	10 weeks	Fiber photometry recordings with social interactions	Involved in data analysis
C1M2	C57BL/6	Female	10 weeks	Fiber photometry recordings with social interactions	AAV virus leakage
C1M3	C57BL/6	Female	10 weeks	Fiber photometry recordings with social interactions	AAV virus leakage
C2M1	C57BL/6	Male	12 weeks	Fiber photometry recordings with social interactions	Involved in data analysis
C3M1	C57BL/6	Male	12 weeks	Fiber photometry recordings with social interactions	AAV virus leakage
C4M1	C57BL/6	Male	12 weeks	Fiber photometry recordings with social interactions	Involved in data analysis
C5M1	C57BL/6	Male	8 weeks	Fiber photometry recordings with social interactions	Involved in data analysis
C5M2	C57BL/6	Male	8 weeks	Fiber photometry recordings with social interactions	Involved in data analysis
C6M1	C57BL/6	Male	8 weeks	Fiber photometry recordings with social interactions	Involved in data analysis
C7M1	C57BL/6	Female	8 weeks	Fiber photometry recordings with social interactions	Involved in data analysis
C7M2	C57BL/6	Female	8 weeks	Fiber photometry recordings with social interactions	Involved in final data
Optrode 1	Thy1::ChR2-EYFP	Male	6 weeks	Optogenetics stimulation with electrophysiological recordings	Device detached at day 21

Optrode 2	Thy1::ChR2-EYFP	Female	6 weeks	Optogenetics stimulation with electrophysiological recordings	Device detached at day 14
Optrode 3	Thy1::ChR2-EYFP	Female	6 weeks	Optogenetics stimulation with electrophysiological recordings	Involved in final data
Optrode 4	Thy1::ChR2-EYFP	Female	6 weeks	Optogenetics stimulation with electrophysiological recordings	Involved in final data
Optrode 5	Thy1::ChR2-EYFP	Female	6 weeks	Optogenetics stimulation with electrophysiological recordings	Involved in final data
43	C57BL/6	Female	12 weeks	Endogenous neural activity recordings	Involved in data analysis
B-5	C57BL/6	Female	12 weeks	Endogenous neural activity recordings	Involved in final data
102	C57BL/6	Female	6 weeks	Immunohistology (1 month) of COMPACT hydrogel and silica fibers	Involved in final data
238	C57BL/6	Male	12 weeks	Immunohistology (1 month) of COMPACT hydrogel and silica fibers	Involved in final data
66	C57BL/6	Male	9 weeks	Immunohistology (1 month) of COMPACT hydrogel and silica fibers	Involved in final data
BIM1	C57BL/6	Male	6 weeks	Immunohistology (14 days) of COMPACT hydrogel and silica fibers	Involved in final data
BIM2	C57BL/6	Male	6 weeks	Immunohistology (14 days) of COMPACT hydrogel and silica fibers	Involved in final data
BIM3	C57BL/6	Male	6 weeks	Immunohistology (14 days) of COMPACT hydrogel and silica fibers	Involved in final data
EBM1	C57BL/6	Male	8 weeks	In vivo incubation of hydrogel microelectrode	Involved in final data
EBM2	C57BL/6	Male	8 weeks	In vivo incubation of hydrogel microelectrode	Involved in final data

Supplementary Reference:

1. Mathot VBF, Pijpers MFJ. Heat capacity, enthalpy and crystallinity of polymers from DSC measurements and determination of the DSC peak base line. *Thermochimica Acta* **151**, 241-259 (1989).

2. Lin S, *et al*. Anti-fatigue-fracture hydrogels. *Science Advances* **5**, eaau8528.

3. Liu X, *et al*. Fatigue-resistant hydrogel optical fibers enable peripheral nerve optogenetics during locomotion. *Nature Methods* **20**, 1802-1809 (2023).
