# Supplementary material for: Control of polymers’ amorphous-crystalline transition enables miniaturization and multifunctional integration for hydrogel bioelectronics
Source: Nat Commun. 2024 Apr 25;15:3525. doi: 10.1038/s41467-024-47988-w (PMC11045824; doi:10.1038/s41467-024-47988-w)
Supplement: Supplementary file 3 — Reporting Summary [file 41467_2024_47988_MOESM3_ESM.pdf]

nature portfolio

Corresponding author(s): Siyuan Rao, Qianbin Wang

Last updated by author(s): Apr 17, 2024

Reporting Summary

Nature Portfolio wishes to improve the reproducibility of the work that we publish. This form provides structure for consistency and transparency in reporting. For further information on Nature Portfolio policies, see our Editorial Policies and the Editorial Policy Checklist.

Statistics

For all statistical analyses, confirm that the following items are present in the figure legend, table legend, main text, or Methods section.

n/a	Confirmed	
☐	☒	The exact sample size (*n*) for each experimental group/condition, given as a discrete number and unit of measurement
☐	☒	A statement on whether measurements were taken from distinct samples or whether the same sample was measured repeatedly
☐	☒	The statistical test(s) used AND whether they are one- or two-sided<br>*Only common tests should be described solely by name; describe more complex techniques in the Methods section.*
☒	☐	A description of all covariates tested
☐	☒	A description of any assumptions or corrections, such as tests of normality and adjustment for multiple comparisons
☐	☒	A full description of the statistical parameters including central tendency (e.g. means) or other basic estimates (e.g. regression coefficient) AND variation (e.g. standard deviation) or associated estimates of uncertainty (e.g. confidence intervals)
☐	☒	For null hypothesis testing, the test statistic (e.g. *F*, *t*, *r*) with confidence intervals, effect sizes, degrees of freedom and *P* value noted<br>*Give P values as exact values whenever suitable.*
☒	☐	For Bayesian analysis, information on the choice of priors and Markov chain Monte Carlo settings
☒	☐	For hierarchical and complex designs, identification of the appropriate level for tests and full reporting of outcomes
☒	☐	Estimates of effect sizes (e.g. Cohen's *d*, Pearson's *r*), indicating how they were calculated

Our web collection on statistics for biologists contains articles on many of the points above.

Software and code

Policy information about availability of computer code

Data collection	-We employed an unbiased algorithm (DeepLabCut) for the analysis of mouse social interactions during fiber photometry recordings, utilizing markerless pose estimation.<br>-We conducted endogenous electrophysiological activity recordings, filtered within the frequency range of 300–3000 Hz and digitized at a sampling frequency of 40 kHz (PowerLab 4/20T, ADInstruments).<br>-We performed light evoked electromyographic and electrophysiological recordings through an amplifier (DAM50, World Precision Instruments) and digitized at 10 kHz (DI-1100, DATAQ Instruments).
Data analysis	Statistical analyses were performed in GraphPad Prism 10 and OriginPro 2022.<br>-For independent data (equal or over 3 groups), we used one-way ANOVA with Tukey’s multiple comparison test.<br>-For independent data (2 groups), we used two-tailed unpaired student's t-test.<br>-For paired data (2 groups), we used two-tailed paired student's t-test.<br>-For hydrogel fiber stability assessments, we used two-way ANOVA with Tukey’s multiple comparison test.<br>-For endogenous spiking activities, signal processing and analysis were conducted using ROSS Offline Spike Sorter.

For manuscripts utilizing custom algorithms or software that are central to the research but not yet described in published literature, software must be made available to editors and reviewers. We strongly encourage code deposition in a community repository (e.g. GitHub). See the Nature Portfolio guidelines for submitting code & software for further information.

Data

Policy information about availability of data

All manuscripts must include a data availability statement. This statement should provide the following information, where applicable:

- Accession codes, unique identifiers, or web links for publicly available datasets
- A description of any restrictions on data availability
- For clinical datasets or third party data, please ensure that the statement adheres to our policy

The data relevant to this study including the material characterizations, biocompatibility tests, fiber photometry recording results, and electrophysiological recording results are comprehensively detailed within the article and its Supplementary Information. Additionally, the original datasets have been made publicly accessible via the public repository figshare (https://figshare.com) and are available here: https://doi.org/10.6084/m9.figshare.25521286.

Research involving human participants, their data, or biological material

Policy information about studies with human participants or human data. See also policy information about sex, gender (identity/presentation), and sexual orientation and race, ethnicity and racism.

Reporting on sex and gender	Not applicable.
Reporting on race, ethnicity, or other socially relevant groupings	Not applicable.
Population characteristics	Not applicable.
Recruitment	Not applicable.
Ethics oversight	Not applicable.

Note that full information on the approval of the study protocol must also be provided in the manuscript.

Field-specific reporting

Please select the one below that is the best fit for your research. If you are not sure, read the appropriate sections before making your selection.

[x] Life sciences [ ] Behavioural & social sciences [ ] Ecological, evolutionary & environmental sciences

For a reference copy of the document with all sections, see nature.com/documents/nr-reporting-summary-flat.pdf

Life sciences study design

All studies must disclose on these points even when the disclosure is negative.

Sample size

Sample sizes are based on values from published literature. For example, the number of samples for fiber characterization is n=4-6, which is determined by power analysis (a = 0.05, p = 0.9). The sample size for fiber photometry recording and social interaction is n=8, similar with other studies (Salinas et al., 2023, Nat. Comm and Cristina et al., 2016 Nat. Methods) of same brain circuits (VTA). The sample size for concurrent optogenetics stimulation and electrophysiological recordings is n=5, which is also similar with other studies (Andres et al., 2015, Nat. Biotech and Seongjun et al., 2021, Nat. Comm). Numbers of samples are stated in the corresponding figure legends and methods section.

Characterization of COMPACT hydrogel materials (membranes, fibers, and cylinders). 4-6 independent samples.

Characterization of COMPACT hydrogel optical fibers. 4 independent samples.

In vivo implantation of COMPACT hydrogel optical fibers: C57BL/6 mice (n = 11).

In vivo implantation of COMPACT hydrogel electrode bundles: C57BL/6 mice (n=2).

In vivo Implantation of COMPACT hydrogel optrode devices: Thy1::ChR2-EYFP mice (n=5).

Implantation of COMPACT hydrogel fibers (n=6) and silica fibers (n=6) in mouse for immune response (30 days, n=3 mice).

Implantation of COMPACT hydrogel fibers (n=3) and silica fibers (n=3) in mouse for immune response (14 days, n=3 mice).

Implntation of COMPACT hydrogel microelectrodes (n=14) in mouse (n=2 mice) for impedance stability studies.

Social behavioral Ttests with fiber photometry recordings:
C57BL/6 mice (n=8) implanted with COMPACT hydrogel optical fibers underwent social behavior tests while simultaneous fiber photometry recordings were conducted between weeks 4 and 10 post-surgery.

	Endogenous spiking activity recordings in mice:<br>Endogenous spiking activity was recorded in C57BL/6 mice (n=2) implanted with COMPACT hydrogel electrode bundles. Recordings were conducted under isoflurane anesthesia, 3 days post-surgery.<br><br>Optogenetic stimulations and electrophysiological recordings in mice:<br>Thy1::ChR2-EYFP mice (n=5) implanted with COMPACT hydrogel optrode devices underwent optogenetic stimulations (under anesthesia) until 10 weeks following their surgeries.
Data exclusions	-3 C57BL/6 mice (implanted with COMPACT hydrogel optical fibers) were excluded because of AAV virus leakage.<br>-2 Thy1::ChR2-EYFP mice (implaned with COMPACT optrode device) were excluded because of device detached in the second week post surgery.
Replication	All attempts at replication were successful for the experiments are listed.<br><br>-During the social behavioral experiments, each test mouse underwent 2 social interaction sessions per week, involving 3 social interactions per session, concurrent with fiber photometry recordings.<br><br>-For endogenous spiking activity recordings, each mouse participated in 2recording sessions per week, with each session consisting of 10 repeated recordings..<br><br>-In the optogenetic stimulation studies, each test mouse was implanted with 3 distinct electrodes. Each electrode was assessed 3 times using electromyographic recordings.<br><br>-Optogenetic stimulation experiments involved electrophysiological measurements on each mouse at week 0, week 1, week 2, week 4, and week 6.<br><br>-In the assessment of hydrogel fiber dimensions, each fiber underwent 9 separate measurements.
Randomization	Randomization is not applicable to this study because different mice (C57BL/6 and Thy1::ChR2-EYFP) were known throughout the experiments and analysis.
Blinding	A separate naive group of blinded investigators conducted data analysis for social interaction analysis. The investigators were not blinded during social behavioral data collection and optogenetic stimulations.<br>For social behavioral data collection, the study goal is to validate the optical stimulation and fiber photometry recording of the hydrogel fibers during social interactions, the investigators were not blinded during optical stimulation and fiber photometry recording nor behavior data collection.<br>For optogenetics stimulation, the study goal is to validate the concurrent light delivery and electrical recording functionality of the optrode devices, the investigators were not blinded during optogenetics stimulation.

Reporting for specific materials, systems and methods

We require information from authors about some types of materials, experimental systems and methods used in many studies. Here, indicate whether each material, system or method listed is relevant to your study. If you are not sure if a list item applies to your research, read the appropriate section before selecting a response.

Materials & experimental systems

n/a	Involved in the study	
	x	Antibodies
	x	Eukaryotic cell lines
x		Palaeontology and archaeology
	x	Animals and other organisms
x		Clinical data
x		Dual use research of concern
x		Plants

Methods

n/a	Involved in the study	
x		ChIP-seq
x		Flow cytometry
x		MRI-based neuroimaging

Antibodies

Antibodies used	Primary antibodies: GFAP (Rabbit, Agilent Dako Z0334, 1:400)<br>GFAP (Mouse, Southern Biotech 12075-01, 1:1000)<br>IBA1 (Rabbit, Invitrogen PA5-119231, 1:400 or 1: 300)<br>CD68 (Rat, Invitrogen 14-0681-82, 1:500)<br>CD16/32 (Rat, Invitrogen 14-0161-82, 1:200)<br>NeuN (Rabbit, Invitrogen PA5-78499, 1:200)<br>Secondary antibodies:<br>Donkey anti-Rabbit (Alexa Fluor 488 Invitrogen A-21206, 1: 200)

	Goat anti-Mouse (Alexa Fluor 555 Invitrogen A-21422, 1:500)<br>Chicken anti-Rabbit (Alexa Fluor 594 Invitrogen A-21442, 1:200)<br>Chicken anti-Rabbit (Alexa Fluor 488 Invitrogen A-21441, 1:200)<br>Goat anti-Rat (Alexa Fluor 555 Invitrogen A-21434, 1:1000)
Validation	Manufactures' website and references from https://www.citeab.com/ were used.<br><br>GFAP (Mouse, Southern Biotech 12075-01, 1:1000):<br>https://www.southernbiotech.com/mouse-anti-human-gfap-unlb-sb61b-12075-01<br><br>IBA1 (Rabbit, Invitrogen PA5-119231, 1:400):<br>https://www.thermofisher.com/antibody/product/IBA1-Antibody-Polyclonal/PA5-119231<br><br>IBA1 (Rabbit, Invitrogen PA5-119231, 1:300):<br>https://www.thermofisher.com/antibody/product/IBA1-Antibody-Polyclonal/PA5-119231<br><br>CD68 (Rat, Invitrogen 14-0681-82, 1:500):<br>https://www.thermofisher.com/antibody/product/CD68-Antibody-clone-FA-11-Monoclonal/14-0681-82<br><br>CD16/32 (Rat, Invitrogen 14-0161-82, 1:200):<br>https://www.thermofisher.com/antibody/product/CD16-CD32-Antibody-clone-93-Monoclonal/14-0161-82<br><br>NeuN (Rabbit, Invitrogen PA5-78499, 1:200) :<br>https://www.thermofisher.com/antibody/product/NeuN-Antibody-Polyclonal/PA5-78499<br><br>Secondary antibodies:<br>Donkey anti-Rabbit (Alexa Fluor 488 Invitrogen A-21206, 1: 200):<br>https://www.thermofisher.com/antibody/product/Donkey-anti-Rabbit-IgG-H-L-Highly-Cross-Adsorbed-Secondary-Antibody-Polyclonal/A-21206<br><br>Goat anti-Mouse (Alexa Fluor 555 Invitrogen A-21422, 1:500):<br>https://www.thermofisher.com/antibody/product/Goat-anti-Mouse-IgG-H-L-Cross-Adsorbed-Secondary-Antibody-Polyclonal/A-21422<br><br>Chicken anti-Rabbit (Alexa Fluor 594 Invitrogen A-21442, 1:200):<br>https://www.thermofisher.com/antibody/product/Chicken-anti-Rabbit-IgG-H-L-Cross-Adsorbed-Secondary-Antibody-Polyclonal/A-21442<br><br>Chicken anti-Rabbit (Alexa Fluor 488 Invitrogen A-21441, 1:200):<br>https://www.thermofisher.com/antibody/product/Chicken-anti-Rabbit-IgG-H-L-Cross-Adsorbed-Secondary-Antibody-Polyclonal/A-21441<br><br>Goat anti-Rat (Alexa Fluor 555 Invitrogen A-21434, 1:1000):<br>https://www.thermofisher.com/antibody/product/Goat-anti-Rat-IgG-H-L-Cross-Adsorbed-Secondary-Antibody-Polyclonal/A-21434

Eukaryotic cell lines

Policy information about cell lines and Sex and Gender in Research

Cell line source(s)	The HEK 293T cell line was a gift from F. Zhang (MIT) and P. Anikeeva (MIT). Detailed information of the cell line can be found here from American Type Culture Collection (item number CRL-3216).
Authentication	HEK 293T cells were authenticated before receiving.
Mycoplasma contamination	The HEK293T cells have not been tested for Mycoplasma contamination after receiving.
Commonly misidentified lines<br>(See ICLAC register)	No commonly misidentified cell lines were used in this study.

Animals and other research organisms

Policy information about studies involving animals; ARRIVE guidelines recommended for reporting animal research, and Sex and Gender in Research

Laboratory animals	C57BL/6 and Thy1::ChR2-EYFP mice (adult mice, 6-8 weeks old) were purchased from the Jackson Laboratory. C57BL/6 and Thy1::ChR2-EYFP mice were bred by UMass Amherst and Binghamton University. Thy1::ChR2-EYFP-homo male and C57BL/6 female were genetically crossed to breed Thy1::ChR2-EYFP-het mice.
Wild animals	No wild animals were used in the study.

Reporting on sex	Both sexes
Field-collected samples	No field collected samples were used in the study.
Ethics oversight	UMass Amherst American Association for Laboratory Animal Science (IACUC) and Binghamton University American Association for Laboratory Animal Science (IACUC).

Note that full information on the approval of the study protocol must also be provided in the manuscript.

Plants

Seed stocks	Not applicable.
Novel plant genotypes	Not applicable.
Authentication	Not applicable.
